# Comparative efficacy and safety of noninvasive respiratory support in very preterm infants (<32 weeks' gestation): a network meta-analysis

**DOI:** 10.3389/fped.2026.1928134

**Published:** 2026-10-28

**Authors:** Shaojuan Huo, Fan Wang, Yayun Zhao, Lixia Chai, Xiaohua He, Jinqiao Guo, Xiaobo Yang, Zhuoyan Li, Xinman Dou

**Affiliations:** 1 Department of Neonatology, Lanzhou University Second Hospital, Lanzhou, China; 2 Department of Pediatric Respiratory Medicine, Lanzhou University Second Hospital, Lanzhou, China; 3 Department of Nursing, Lanzhou University Second Hospital, Lanzhou, China

**Keywords:** very preterm infants, noninvasive ventilation, NCPAP, NIPPV, NHFOV, HFNC, NIV-NAVA, network meta-analysis

## Abstract

**Background:**

The optimal noninvasive respiratory support strategy for very preterm infants among NCPAP, NIPPV, NHFOV, HFNC, and NIV-NAVA remains unclear.

**Methods:**

This network meta-analysis of nine randomized controlled trials involving 1,449 preterm infants compared the efficacy and safety of these five modes evaluated in eight trials of early respiratory support and one trial of support after clinical stabilization.

**Results:**

The primary efficacy outcomes were treatment failure or endotracheal intubation, bronchopulmonary dysplasia (BPD), and mortality; safety outcomes included retinopathy of prematurity, patent ductus arteriosus, necrotizing enterocolitis (NEC), intraventricular hemorrhage (IVH), and air leak syndrome. NIPPV (RR = 0.47, 95% CI 0.34 to 0.65) and NHFOV (RR = 0.62, 95% CI 0.43 to 0.91) were associated with lower risks of treatment failure or intubation than NCPAP, and NIPPV showed a lower risk than HFNC (RR = 0.53, 95% CI 0.29 to 0.98). The latter difference was not maintained after exclusion of the trial evaluating support after stabilization. No significant differences were observed in BPD or mortality. NIPPV was associated with a lower risk of NEC than NCPAP (RR = 0.60, 95% CI 0.37 to 0.97), although differences in severity thresholds make this finding exploratory. No significant differences were found for the other safety outcomes, including IVH.

**Conclusions:**

These findings suggest potential respiratory benefits without establishing equivalent efficacy or safety. Interpretation is limited by differences in outcome definitions and clinical settings; assessable comparisons were provisionally rated as low or very low certainty. Applicability specifically to infants born before 32 weeks remains uncertain.

**Systematic Review Registration:**

https://www.crd.york.ac.uk/PROSPERO/view/CRD420261403266, PROSPERO CRD (420261403266).

## Introduction

Very preterm infants (gestational age <32 weeks) are among the most critically ill patients in the neonatal intensive care unit (NICU). Due to pulmonary immaturity, surfactant deficiency, and immature respiratory control, these infants are highly susceptible to respiratory distress syndrome immediately after birth and often require prompt respiratory support (1, 2). Although invasive mechanical ventilation can effectively improve gas exchange, prolonged or repeated intubation in these infants is associated with complications including bronchopulmonary dysplasia and neurodevelopmental impairment (3, 4). Therefore, clinical guidelines recommend the early use of noninvasive respiratory support in the absence of contraindications to avoid invasive ventilation and its associated harms whenever possible (5).

Noninvasive respiratory support strategies used in preterm infants include nasal continuous positive airway pressure (NCPAP), a widely used mode that may reduce bronchopulmonary dysplasia or death compared with routine initial intubation and mechanical ventilation (6). Nasal intermittent positive pressure ventilation (NIPPV) superimposes preset positive pressure pulses on NCPAP and may reduce treatment failure or intubation, although evidence specifically for infants born before 32 weeks remains limited. NIPPV can be synchronized with the infant's inspiratory effort or delivered without synchronization, a distinction that may influence treatment effects (7). Nasal high-frequency oscillatory ventilation (NHFOV) combines high-frequency oscillations with continuous positive airway pressure to facilitate gas exchange, but evidence of reduced intubation remains dependent on the population and clinical setting (8). High-flow nasal cannula (HFNC) provides heated, humidified gas and may reduce nasopharyngeal dead space and the work of breathing. It has been associated with less nasal injury than NCPAP, although this does not establish comparable efficacy in extremely preterm infants (9). Noninvasive neurally adjusted ventilatory assist (NIV-NAVA) triggers ventilation via diaphragmatic electrical activity, aligning support with inspiratory effort, but evidence of reduced treatment failure remains limited (10). These noninvasive ventilation modes differ in physiological mechanisms, control strategies, and device requirements, which may affect their suitability and effectiveness across clinical settings.

Within the very preterm population, the evidence is uneven across gestational ages, with particularly limited data for extremely preterm infants born before 32 weeks (11). Pairwise meta-analyses provide evidence for individual comparisons, but a network meta-analysis can integrate available direct and indirect evidence to compare multiple strategies. Such comparisons require careful consideration of differences in gestational age, clinical indication, and intervention delivery, including NIPPV synchronization (7, 12).

This network meta-analysis was conducted to compare the efficacy and safety of NCPAP, NIPPV, NHFOV, HFNC, and NIV-NAVA in very preterm infants born before 32 weeks receiving noninvasive respiratory support. The findings were interpreted in relation to the gestational-age populations and clinical settings represented in the included trials.

## Methods

### Data sources

This study was reported with reference to the PRISMA 2020 guidelines (13) and the PRISMA-NMA extension (14) and registered with PROSPERO (CRD420261403266).

### Literature inclusion and exclusion criteria

This systematic review and network meta-analysis included randomized controlled trials (RCTs) enrolling very preterm infants born before 32 weeks receiving noninvasive respiratory support, either as early support or after stabilization. Data were extracted from the overall eligible trial populations. The interventions compared were NCPAP, NIPPV, NHFOV, HFNC, and NIV-NAVA. Efficacy outcomes included treatment failure or endotracheal intubation, with assessment time points identified for each study, bronchopulmonary dysplasia, and mortality. Safety outcomes included retinopathy of prematurity (ROP), patent ductus arteriosus (PDA), necrotizing enterocolitis (NEC), intraventricular hemorrhage (IVH), air leak outcomes, and other adverse events. Outcomes were recorded according to each trial's diagnostic criteria, severity thresholds, and assessment periods.

Studies were excluded if they were duplicate publications, non-English articles, reviews, comments, or animal studies; if the full text was unavailable, including conference abstracts without a full report; if data could not be extracted or transformed from the original study; or if they were single-arm studies. Trials comparing sustained inflation during initial resuscitation, invasive ventilation modes, or different withdrawal schedules for the same CPAP modality were excluded. Biphasic CPAP was not classified as NCPAP, and trials requiring this equivalence to enter the network were excluded. Intervention classification was based on the respiratory mode and interface used in each randomized arm. Synchronized and nonsynchronized NIPPV were grouped within one node on the basis of their shared delivery of intermittent positive-pressure assistance, with synchronization status recorded separately. HFNC and HHHFNC were grouped when both described heated, humidified high-flow support through nasal cannulae. Noninvasive support triggered by diaphragmatic electrical activity was consistently labelled NIV-NAVA.

### Literature search strategy

We systematically searched PubMed, Cochrane Library, Embase, Web of Science, and CINAHL for published RCTs. The search combined terms for prematurity, noninvasive respiratory support, and randomized trials, including “Premature Infant,” “Premature Birth,” “very preterm,” “very low birth weight,” “extremely low birth weight,” “noninvasive ventilation,” “continuous positive airway pressure,” “nasal intermittent positive pressure ventilation,” “high-frequency oscillatory ventilation,” “high-flow nasal cannula,” and “neurally adjusted ventilatory assist,” together with relevant abbreviations. HHHFNC denotes heated humidified high-flow nasal cannula. Two researchers independently conducted the search using a combination of subject headings and free-text words. The search was originally conducted from database inception to March 19, 2026, and was repeated before submission; no additional eligible studies were identified. We also manually screened the reference lists of relevant systematic reviews, meta-analyses, and included studies. The detailed PubMed search strategy is provided in Supplementary Table S1.

### Literature screening and data extraction

EndNote 21 software was used for reference management. Two reviewers independently screened the literature and extracted data according to the eligibility criteria. Study eligibility was reassessed during revision. Any disagreements were resolved by consulting a third reviewer with neonatal clinical expertise. The extracted data included first author, year of publication, gestational age, birth weight, sample size, interventions, and outcome measures. Group-specific baseline values were retained in their original reporting format, distinguishing means and standard deviations from medians, interquartile ranges, and ranges. Additional eligibility and extraction details included gestational-age entry criteria, clinical setting at randomization, previous respiratory support, reported NIPPV synchronization, and arm-specific event counts and denominators. For each outcome, diagnostic criteria, severity thresholds, and assessment periods were recorded alongside the corresponding denominator. Extraction distinguished escalation to another noninvasive mode from endotracheal intubation, and recorded mortality according to the reported follow-up period. Study-specific definitions are summarized in the supplementary table of study-specific outcome definitions and assessment periods, with unspecified details explicitly identified. Characteristics relevant to transitivity, including country, recruitment period, gestational age, birth weight, antenatal corticosteroid exposure, clinical setting, and surfactant strategy, are summarized by randomized comparison in Supplementary Table A1.

### Quality assessment of included studies

Two reviewers independently assessed risk of bias using the original Cochrane risk-of-bias tool (RoB 1), with assessments recorded in RevMan 5.4.1 (15). Disagreements were resolved through discussion or consultation with a third reviewer. The assessment covered random sequence generation, allocation concealment, blinding of participants and personnel, blinding of outcome assessment, incomplete outcome data, selective reporting, and other sources of bias. Each domain was rated as “low risk,” “high risk,” or “unclear risk.” No numerical scores or A/B/C classifications were assigned. Blinding-related judgments considered the susceptibility of the outcome to knowledge of treatment allocation. Lack of blinding alone was not considered sufficient to indicate high risk for mortality, whereas intubation decisions could be influenced by clinical discretion and the application of escalation criteria. Risk of bias was distinguished from certainty of evidence, which was considered separately using CINeMA.

### Certainty of evidence

Evidence profiles were prepared for each comparison and outcome using the CINeMA framework, covering within-study bias, reporting bias, indirectness, imprecision, heterogeneity, and incoherence. These assessments were distinguished from study-level risk-of-bias judgments. Where differing or insufficiently specified outcome definitions prevented a clinically coherent interpretation, the overall rating was left unassigned. These entries were labelled “Not assessable” in Supplementary Table and were not treated as a CINeMA confidence category.

### Statistical analysis

The primary network analyses were performed using Stata 19.0. The outcome measures were binary variables, and treatment effects were estimated using the relative risk (RR) with its 95% confidence interval (CI). RRs were selected to express relative event probabilities directly. An RR below 1 favored the first treatment in each comparison. A 95% CI that did not include 1 was considered statistically significant, whereas an interval including 1 did not establish equivalence. All outcome networks and treatment rankings were recalculated using the revised study set, with no contribution from excluded trials. Synchronized and nonsynchronized NIPPV remained grouped within the NIPPV node, and this variation was considered when interpreting the pooled estimates. Estimates combining differing or incompletely specified outcome definitions were considered exploratory and were not interpreted as effects on a uniform severity-specific outcome. The surface under the cumulative ranking curve (SUCRA) was used to rank the interventions, with a higher SUCRA value indicating a more favorable ranking for reducing the adverse outcome assessed. A league table summarized the pairwise comparisons. Comparison-adjusted funnel plots were examined descriptively for small-study effects. As each outcome included fewer than 10 independent trials, Egger test results were not used to infer the presence or absence of publication bias or the robustness of the findings. Multiple comparisons from the same trial were not counted as independent studies (16).

A frequentist multivariate random-effects model was fitted to log RRs, with a common between-study variance estimated by restricted maximum likelihood within each outcome network. Correlations between contrasts from multi-arm trials were accounted for through the shared reference arm, with random-effects variances and covariances specified as *τ*^2^ and *τ*^2^/2, respectively.

When an arm contained zero events, 0.5 was added to the event and nonevent counts in every arm of that trial. Trials with no events in any arm were retained in descriptive summaries but omitted from the main supplementary log-RR model. Sensitivity analyses examined alternative continuity corrections and retention of all-zero trials. Heterogeneity was summarized using *τ*^2^ and total Q-based I^2^. Exploratory 95% prediction intervals incorporated the estimated between-study variance but did not account for uncertainty in *τ*^2^. Global incoherence was assessed by comparing consistency and design-specific models conditional on the estimated *τ*^2^. Local checks compared direct evidence with indirect estimates obtained after excluding all directly comparing trials. Non-estimable checks were identified explicitly, and nonsignificant tests were not interpreted as evidence of coherence. *Post hoc* sensitivity analyses examined a common-effect model and separate exclusion of ENTARES, Zhang 2026, and the four-arm Öktem trial. ENTARES was excluded to assess the influence of support after stabilization; this *post hoc* analysis did not constitute a comparison between primary and postextubation support. Only one trial evaluated support after stabilization, and a sensitivity analysis was performed.

## Results

### Literature search and screening results

A total of 17,896 records were identified through the initial search. After removing 8,030 duplicate records using EndNote 21 software and manual screening, 9,866 records remained. Following screening of titles and abstracts, 9,687 records were excluded, leaving 179 records for full-text assessment. Of these, 170 were excluded and 9 studies were included in the review (Figure 1).

**Figure 1:**
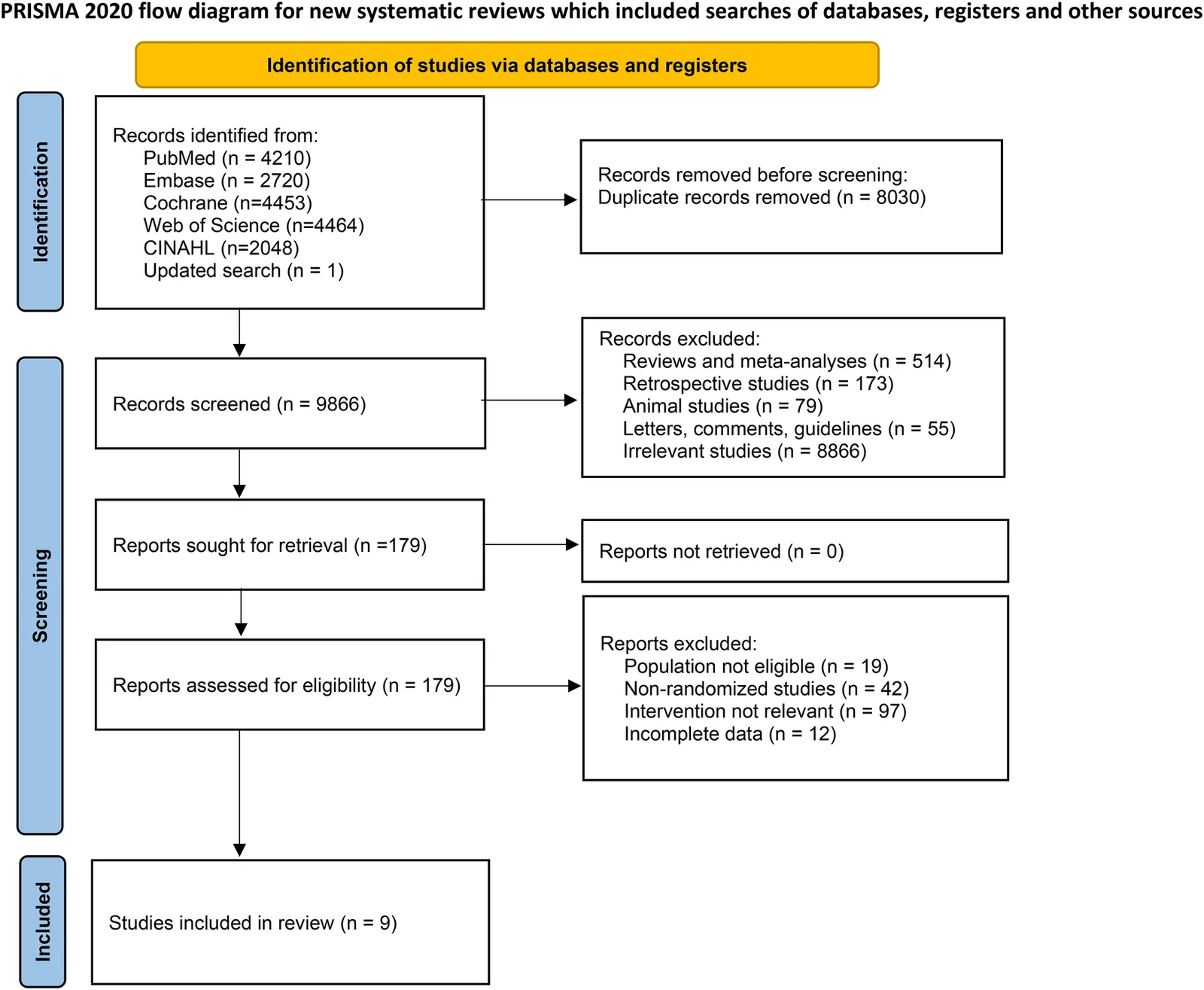
PRISMA flow diagram of the study selection process.

### Characteristics and quality assessment of the included studies

A total of 9 randomized controlled trials (17–25), comprising 1,449 infants in the reported analysis populations, were included. Five noninvasive ventilation modes were involved: NCPAP, NIPPV, NHFOV, HFNC, and NIV-NAVA. The trials enrolled broader preterm populations rather than exclusively infants born before 32 weeks. Data were extracted from the reported overall trial populations without restriction to a separate <32-week subgroup. Eight evaluated early respiratory support, whereas ENTARES enrolled infants after at least 48 h of stability on noninvasive support (17). The baseline characteristics of the included studies are presented in Table 1. Additional characteristics relevant to transitivity, including recruitment period, country, antenatal corticosteroid exposure, and surfactant strategy, are summarized in Supplementary Table A1. Diagnostic criteria, severity thresholds, and follow-up periods differed across trials, and some reports did not specify these details. Additional adverse events reported by individual trials are summarized in Table 2.

**Table 1:** Characteristics of the included randomized controlled trials.

Study	Country	Single/multi-center	Ventilation mode and equipment parameters	Enrolment period	Gestational age (weeks)	Birth weight (g)	Intervention	Outcomes	Definitions of extracted BPD, PDA, and air-leak outcomes	Risk of bias^1^
Gharehbaghi 2019 (20) (*n* = 61)	Iran	Single-center	NIPPV: Inspiration 5i ventilator, short bilateral nasal prongs, PEEP 5–6 cmH₂O, PIP 18–20 cmH₂O, rate 30–40/min(synchronized mode). NCPAP: Fisher & Paykel variable-flow device, short bilateral nasal prongs, PEEP 5–6 cmH₂O.	January 2017-September 2017	30.4 ± 1.3/29.8 ± 1.7ᵃ	1,301 ± 167/1,246 ± 201ᵃ	NIPPV (*n* = 30)/NCPAP (*n* = 31)	①②③④⑤⑥⑦⑧	BPD: supplemental oxygen or mechanical respiratory support through day 28. PDA: diagnostic threshold NS. Air leak: radiographically diagnosed pneumothorax.	Unclear: AC, PB, OB
Dursun 2019 (19) (*n* = 84)	Turkey	Single-center	nIPPV: SLE 5,000 ventilator, short binasal prongs, PEEP 6-8 cmH₂O, PIP 16-20 cmH₂O, rate 30-40/min (synchronized mode). nCPAP: SLE 2,000 ventilator, short binasal prongs, PEEP 6-8 cmH₂O.	November 2013-November 2014	29.2 ± 1.7/29.4 ± 1.5ᵃ	1,356 ± 295/1,359 ± 246ᵃ	Synchronized NIPPV (*n* = 42)/NCPAP (*n* = 42)	①②③④⑥⑦⑧	BPD: NIH consensus criteria; extracted severity threshold NS. PDA: not included in the analysis dataset. Air leak: pneumothorax.	Unclear: PB
Öktem 2021 (23) (*n* = 76)	Turkey	Single-center	HHHFNC: Vapotherm precision-flow device, small-bore cannula, initial flow 5 L/min. NCPAP: Fisher & Paykel bubble CPAP system, short binasal prongs, PEEP 5-6 cmH₂O. NIPPV: Drager Babylog 8,000 ventilator, short binasal prongs, PEEP 5-6 cmH₂O, PIP 15-20 cmH₂O, rate 25-30/min (non-synchronized mode). NHFOV: Drager Babylog 8,000 high-frequency oscillatory ventilator, short binasal prongs, MAP 6 cmH₂O, rate 10 Hz.	December 2015-February 2017	28 (26 to 32)/28 (26 to 32)/28 (26 to 32)/29 (27 to 32)ᶜ	1,190 (600 to 2,010)/1,240 (580 to 2,010)/1,130 (530 to 2,550)/1,250 (800 to 2,240)ᶜ	HFNC (*n* = 20)/NCPAP (*n* = 20)/Nonsynchronized NIPPV (*n* = 19)/NHFOV (*n* = 17)	①②③⑤⑥⑦⑧	BPD: diagnostic criteria and assessment age NS. PDA: diagnostic threshold NS. Air leak: air-leak syndrome; components NS.	Unclear: AC, PB, OB
Cresi 2023 (17) (*n* = 247)	Italy	Multi-center	NCPAP: initial PEEP 5-7 cmH₂O. HHHFNC: initial flow 4-7 L/min.	November 1, 2018-June 30, 2021	28 (27 to 29)/28 (27 to 29)ᵇ	1,054 ± 220/1,085 ± 243ᵃ	NCPAP (*n* = 122)/HFNC (*n* = 125)	①②③④⑤⑥⑦⑧	BPD: oxygen requirement at 36 weeks’ PMA or 28 days of life, as reported. PDA: requiring treatment. Air leak: pneumothorax.	Unclear: PB
Demirel 202 (18) (*n* = 107)	Turkey	Single-center	HFNC: Vapotherm device, initial flow 6 L/min, maximum up to 8 L/min. nCPAP: SERVO-i ventilator, short binasal prongs, initial PEEP 6 cmH₂O, maximum up to 7 cmH₂O.	February 2017-February 2018	31.2 (2.3)/31.0 (1.8)ᵈ	1,570 (455)/1,505 (580)ᵈ	HFNC (*n* = 53)/NCPAP (*n* = 54)	①②③④⑤⑥⑦⑧	BPD: diagnostic criteria and assessment age NS. PDA: diagnostic threshold NS. Air leak: pneumothorax.	Unclear: PB
Oncel 2016 (24) (*n* = 200)	Turkey	Single-center	NCPAP: SLE ventilator, short binasal prongs, PEEP 5-6 cmH₂O; NIPPV: SLE ventilator, short binasal prongs,PEEP 5-6 cmH₂O, PIP 15-20 cmH₂O, rate 20-30/min（non-synchronized mode).	November 2012-November 2013	29.1 ± 1.6/29.2 ± 1.7ᵃ	1,175 ± 214/1,180 ± 206ᵃ	NCPAP (*n* = 100)/Nonsynchronized NIPPV (*n* = 100)	①②③④⑤⑥⑦⑧	BPD: moderate-to-severe disease according to NIH criteria, reported among survivors to discharge. PDA: medication and surgical ligation reported separately. Air leak: pneumothorax.	Unclear: PB, DB
Zhang 2026 (25) (*n* = 312)	China	Multi-center	NCPAP: nasal prongs, PEEP 6-8 cmH₂O. NIPPV: nasal prongs, PEEP 6-8 cmH₂O, PIP 15-20 cmH₂O, rate 20-40/min（synchronized mode).	December 2021-October 2024	28.5 (27.6 to 29.3)/29.0 (28.1 to 29.4)ᵇ	1,120 (947.5 to 1,270)/1,130 (950 to 1,310)ᵇ	NCPAP (*n* = 153)/Synchronized NIPPV (*n* = 159)	①②③④⑤⑥⑦⑧	BPD: Jensen 2019 criteria based on respiratory support at 36 weeks’ PMA. PDA: hemodynamically significant PDA. Air leak: pneumothorax.	Unclear: PB; high: OB
Lee 2022 (21) (*n* = 20)	Finland	Single-center	NIV-NAVA: Servo-n ventilator, EasyFlow nasal interface, PEEP 6 cmH₂O, NAVA level 1.0 cmH₂O/*μ*V. NCPAP: Servo-n ventilator, EasyFlow nasal interface, PEEP 6 cmH₂O.	January 2017-July 2019	29⁺⁴ ± 2⁺⁰/29⁺⁶ ± 1⁺^2^ᵃ,ᵉ	1,331 ± 370/1,346 ± 379ᵃ	NIV-NAVA (*n* = 10)/NCPAP (*n* = 10)	①⑧	BPD and PDA: not included in the analysis dataset. Air leak: pneumothorax; the reported event followed the allocated intervention and subsequent intubation.	Unclear: PB, ID, OB
Li 2025 (22) (*n* = 342)	China	Multi-center	NHFOV: oscillatory ventilator, short binasal prongs, initial MAP 6 cmH₂O, frequency 10 Hz, amplitude 15 cmH₂O. NCPAP: continuous-flow ventilator, short binasal prongs, initial PEEP 6 cmH₂O.	August 2022-August 2024	27.0 (26.0 to 28.0)/27.0 (26.0 to 28.0)ᵇ	990 (820 to 1,100)/970 (880 to 1,090)ᵇ	NHFOV (*n* = 170)/NCPAP (*n* = 172)	①②③④⑤⑥⑦⑧	BPD: moderate-to-severe disease using the reported 2019 definition. PDA: hemodynamically significant PDA specified in the Methods; outcome table labelled PDA. Air leak: pneumothorax or other air leaks during assigned treatment.	Unclear: PB

Values are presented in the same order as the intervention groups. ᵃMean ± SD. ᵇMedian (25th to 75th percentile). ᶜMedian (minimum to maximum). ᵈMedian (IQR width), as reported in the original article. ᵉGestational age is expressed as weeks⁺days, preserving the original notation.

Outcomes: ①Treatment failure or endotracheal intubation; ②Bronchopulmonary dysplasia; ③Mortality; ④Retinopathy of prematurity; ⑤Patent ductus arteriosus; ⑥Necrotizing enterocolitis; ⑦Intraventricular hemorrhage; ⑧Air leak, including pneumothorax. Outcomes refer to those available for this review, including reported zero events. Definitions, severity thresholds, assessment periods, and outcome-specific denominators varied across studies and are detailed in the supplementary outcome-definition table.

1 Risk-of-bias entries summarize the supplied domain-level assessments; domains not listed were rated as low risk. AC, allocation concealment; PB, blinding of participants and personnel; DB, blinding of outcome assessment; ID, incomplete outcome data; OB, other sources of bias. These entries are not overall certainty ratings and require outcome-specific interpretation.

NCPAP, nasal continuous positive airway pressure; NIPPV, nasal intermittent positive pressure ventilation; NHFOV, nasal high-frequency oscillatory ventilation; HFNC, high-flow nasal cannula; NIV-NAVA, noninvasive neurally adjusted ventilatory assist.

**Table 2:** Additional adverse events reported with different noninvasive respiratory support strategies in the included randomized controlled trials.

A. General adverse events.				
Study	Intervention groups	Reported adverse event(s)	Events, n	Total, n
Öktem 2021 (23)	HFNC/NCPAP/NIPPV/NHFOV	①②	15/15/20/22	20/20/19/17
Li 2025 (22)	NHFOV/NCPAP	①	5/9	170/172

B. Serious adverse events.				
Study	Intervention groups	Reported adverse event(s)	Events, n	Total, n
Gharehbaghi 2019 (20)	NIPPV/NCPAP	②⑥	11/7	30/31
Dursun 2019 (19)	NIPPV/NCPAP	①	6/4	42/42
Öktem 2021 (23)	HFNC/NCPAP/NIPPV/NHFOV	①④	15/13/24/19	20/20/19/17
Cresi 2023 (17)	NCPAP/HFNC	①	39/51	122/125
Demirel 2021 (18)	HFNC/NCPAP	①	6/7	53/54
Oncel 2016 (24)	NCPAP/NIPPV	②③	8/8	100/100
Zhang 2026 (25)	NCPAP/NIPPV	①②⑤	42/34	153/159
Li 2025 (22)	NHFOV/NCPAP	②④	15/12	170/172

① Nasal injury; ② abdominal distension.

① Sepsis; ② pulmonary hemorrhage; ③ spontaneous intestinal perforation; ④ mucus plugging; ⑤ periventricular leukomalacia; ⑥ severe apnea.

NCPAP, nasal continuous positive airway pressure; NIPPV, nasal intermittent positive pressure ventilation; NHFOV, nasal high-frequency oscillatory ventilation; HFNC, high-flow nasal cannula; NIV-NAVA, noninvasive neurally adjusted ventilatory assist.

All nine trials were rated at low risk for random sequence generation, and seven trials were rated at low risk for selective reporting. Allocation concealment was unclear in two trials. Blinding of participants and personnel was rated as unclear in all nine trials, while blinding of outcome assessment and incomplete outcome data were each unclear in one trial. Other sources of bias were rated as unclear in three trials and high risk in one trial (Supplementary Figure 1). These domain-level judgments were not converted into an overall study-quality grade.

### Certainty of evidence

The supplementary evidence profiles contained 56 comparisons across the eight review outcomes and six additional comparisons for severe IVH. For treatment failure or intubation, NIPPV vs. NCPAP and NHFOV vs. NCPAP were provisionally rated as low certainty; the other eight comparisons were provisionally rated as very low certainty. All mortality, air-leak, and severe-IVH comparisons were provisionally rated as very low certainty. Overall ratings remained unassigned for the 30 comparisons involving BPD, ROP, PDA, NEC, and mixed-definition IVH because the extracted outcomes did not represent sufficiently consistent clinical definitions (supplementary CINeMA evidence profiles).

## Results of the network meta-analysis

### Clinical efficacy

#### Treatment failure or endotracheal intubation

##### Evidence network

A total of nine RCTs contributed to the network for treatment failure or endotracheal intubation, involving five noninvasive respiratory support modes. Definitions and assessment periods varied across trials; the pooled outcome therefore did not represent intubation within 72 h alone. In the evidence network plot, each node represents an intervention, and each edge represents an available direct comparison. The network was centered on NCPAP, with all five interventions connected (Figure 2A). A consistency model was used for the network meta-analysis.

**Figure 2:**
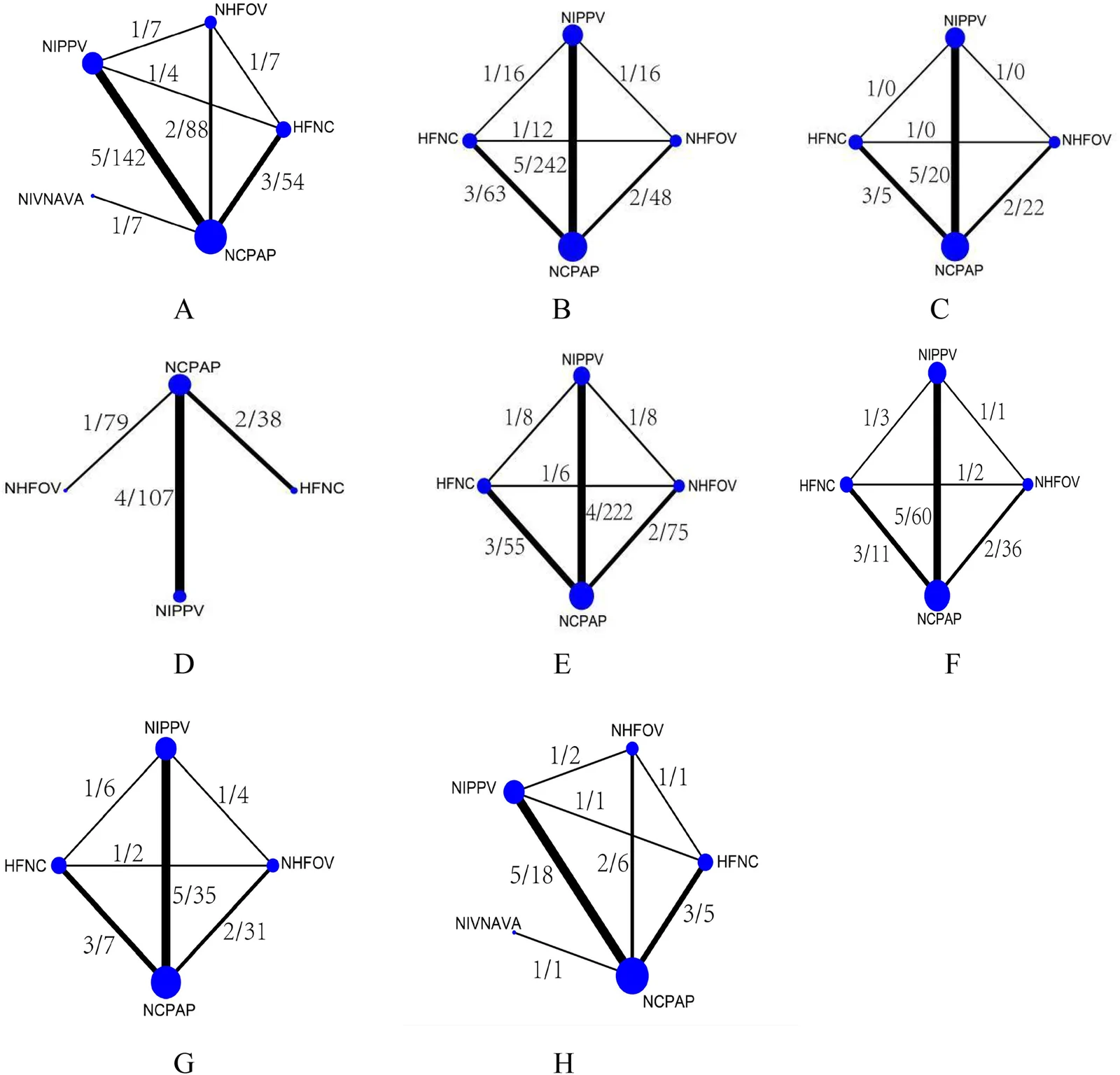
Network plot of clinical effectiveness indicators for different non-invasive ventilation strategies in extremely preterm infants. NCPAP, nasal continuous positive airway pressure; NIPPV, nasal intermittent positive pressure ventilation; NHFOV, nasal high-frequency oscillatory ventilation; HFNC, high-flow nasal cannula; NIV-NAVA, non-invasive neurally adjusted ventilatory assist. In the network plot, node area represents the number of infants, and edge width represents the number of independent trials for direct comparison. Multi-arm trials contribute evidence to multiple pairwise comparisons.

##### Network meta-analysis and SUCRA ranking

The five respiratory support modes generated 10 pairwise comparisons. Compared with NCPAP, NIPPV (RR = 0.47, 95% CI 0.34 to 0.65) and NHFOV (RR = 0.62, 95% CI 0.43 to 0.91) were associated with lower risks of treatment failure or intubation. No significant differences were observed for NIV-NAVA (RR = 0.75, 95% CI 0.22 to 2.52) or HFNC (RR = 0.88, 95% CI 0.53 to 1.48). NIPPV was also associated with a lower risk than HFNC (RR = 0.53, 95% CI 0.29 to 0.98); the remaining comparisons were not statistically significant (Figure 3A). SUCRA values ranked from highest to lowest were NIPPV (90.2%), NHFOV (64.9%), NIV-NAVA (47.0%), HFNC (31.8%), and NCPAP (16.2%). Across outcomes, higher SUCRA values indicated more favorable rankings for reducing the event assessed, but did not establish superiority.

**Figure 3:**
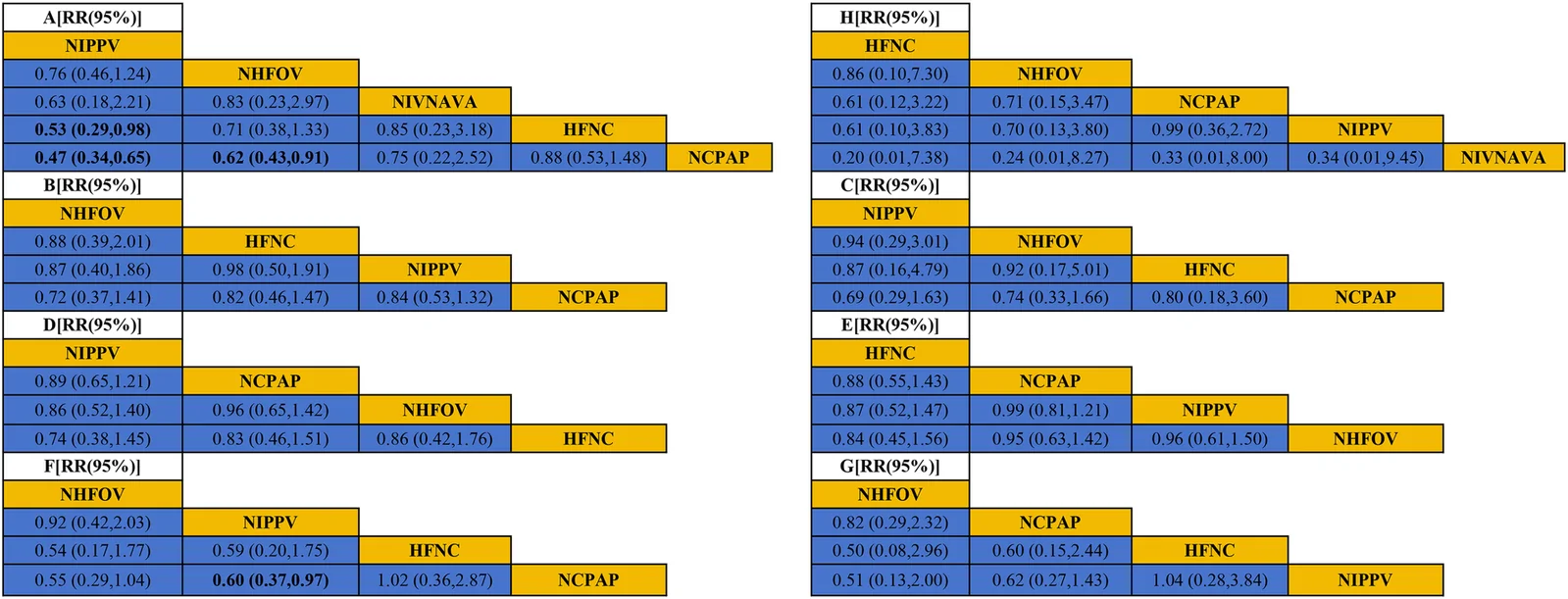
Network meta-analysis plots for each outcome of clinical effectiveness of different non-invasive ventilation strategies in very preterm infants. NCPAP, nasal continuous positive airway pressure; NIPPV, nasal intermittent positive pressure ventilation; NHFOV, nasal high-frequency oscillatory ventilation; HFNC, high-flow nasal cannula; NIV-NAVA, non-invasive neurally adjusted ventilatory assist.

#### Incidence of bronchopulmonary dysplasia

##### Evidence network

A total of eight RCTs reported bronchopulmonary dysplasia, involving four modes: NCPAP, NIPPV, NHFOV, and HFNC. The network contained closed loops (Figure 2B).

##### Network meta-analysis and SUCRA ranking

The four modes generated six pairwise comparisons. Compared with NCPAP, NHFOV (RR = 0.72, 95% CI 0.37 to 1.41), HFNC (RR = 0.82, 95% CI 0.46 to 1.47), and NIPPV (RR = 0.84, 95% CI 0.53 to 1.32) showed no statistically significant differences in bronchopulmonary dysplasia. The remaining comparisons were also not statistically significant (Figure 3B). SUCRA values ranked from highest to lowest were NHFOV (69.1%), HFNC (54.7%), NIPPV (54.3%), and NCPAP (21.9%).

#### Mortality

##### Evidence network

A total of eight RCTs reported mortality, involving four modes: NCPAP, NIPPV, NHFOV, and HFNC. The network contained closed loops (Figure 2C).

##### Network meta-analysis and SUCRA ranking

The four modes generated six pairwise comparisons. Compared with NCPAP, NIPPV (RR = 0.69, 95% CI 0.29 to 1.63), NHFOV (RR = 0.74, 95% CI 0.33 to 1.66), and HFNC (RR = 0.80, 95% CI 0.18 to 3.60) showed no statistically significant differences in mortality. The remaining comparisons were also not statistically significant (Figure 3C). SUCRA values ranked from highest to lowest were NIPPV (64.0%), NHFOV (58.4%), HFNC (50.4%), and NCPAP (27.1%).

### Safety outcomes

#### Incidence of retinopathy of prematurity

##### Evidence network

A total of seven RCTs reported retinopathy of prematurity, involving four modes: NCPAP, NIPPV, NHFOV, and HFNC. The network was centered on NCPAP and contained no closed loops (Figure 2D), precluding assessment of agreement between direct and indirect evidence within this network.

##### Network meta-analysis and SUCRA ranking

The four modes generated six pairwise comparisons. None showed a statistically significant difference in retinopathy of prematurity, with all 95% CIs including 1 (Figure 3D). SUCRA values ranked from highest to lowest were NIPPV (76.6%), NCPAP (51.4%), NHFOV (44.7%), and HFNC (27.3%).

#### Incidence of patent ductus arteriosus

##### Evidence network

A total of seven RCTs reported patent ductus arteriosus, involving four modes: NCPAP, NIPPV, NHFOV, and HFNC. The network contained closed loops (Figure 2E).

##### Network meta-analysis and SUCRA ranking

The four modes generated six pairwise comparisons. No statistically significant differences in patent ductus arteriosus were observed between interventions, with all 95% CIs including 1 (Figure 3E). SUCRA values ranked from highest to lowest were HFNC (69.3%), NCPAP (48.2%), NIPPV (45.0%), and NHFOV (37.4%). These rankings did not establish superiority for reducing PDA.

#### Incidence of necrotizing enterocolitis

##### Evidence network

A total of eight RCTs reported necrotizing enterocolitis, involving four modes: NCPAP, NIPPV, NHFOV, and HFNC. The network contained closed loops (Figure 2F).

##### Network meta-analysis and SUCRA ranking

The four modes generated six pairwise comparisons. Compared with NCPAP, NIPPV was associated with a lower risk of necrotizing enterocolitis (RR = 0.60, 95% CI 0.37 to 0.97). No significant differences were observed for NHFOV (RR = 0.55, 95% CI 0.29 to 1.04) or HFNC (RR = 1.02, 95% CI 0.36 to 2.87). The remaining comparisons were not statistically significant (Figure 3F). Differences in reported NEC severity thresholds limited interpretation of the pooled estimates. SUCRA values ranked from highest to lowest were NHFOV (80.2%), NIPPV (74.0%), HFNC (27.0%), and NCPAP (18.7%).

#### Incidence of intraventricular hemorrhage

##### Network of evidence

A total of eight RCTs involving 1,429 infants reported intraventricular hemorrhage, involving four modes: NCPAP, NIPPV, NHFOV, and HFNC. The network contained closed loops (Figure 2G). Three trials directly compared HFNC with NCPAP, reporting 6/198 and 1/196 events, respectively.

##### Network meta-analysis and SUCRA ranking

The four modes generated six pairwise comparisons. No statistically significant differences in intraventricular hemorrhage were observed between interventions, with all 95% CIs including 1. The previously reported statistically significant increase in IVH risk with HFNC was not reproduced in the revised analysis (Figure 3G). However, the confidence intervals did not exclude clinically important differences, and the pooled outcome combined different IVH severity categories. SUCRA values ranked from highest to lowest were NHFOV (75.5%), NCPAP (66.6%), HFNC (30.5%), and NIPPV (27.3%).

#### Incidence of air leak syndrome

##### Evidence network

A total of nine RCTs reported air leak syndrome, involving five modes: NCPAP, NIPPV, NHFOV, HFNC, and NIV-NAVA. The network contained closed loops (Figure 2H).

##### Network meta-analysis and SUCRA ranking

The five modes generated 10 pairwise comparisons. No statistically significant differences in air leak syndrome were observed between interventions, with all 95% CIs including 1 (Figure 3H). SUCRA values ranked from highest to lowest were HFNC (70.0%), NHFOV (63.6%), NCPAP (46.8%), NIPPV (46.5%), and NIV-NAVA (23.1%).

##### Supplementary analyses

In the independent supplementary analysis, *τ*^2^ was estimated as 0.1094 for BPD, 0.1700 for IVH, and 0.1450 for air leak, and as zero for the remaining outcomes. Total Q-based I^2^ was 38.0% for treatment failure or intubation, 46.5% for BPD, and 2.5% for IVH, and was zero for the other outcomes. These measures describe different aspects of variability and do not establish clinical homogeneity. Prediction intervals are presented in Supplementary Table A5. Eight trials reported mortality, but seven contributed to the supplementary log-RR model because Demirel 2021 reported no deaths in either arm.

Global incoherence tests were not statistically significant where estimable. Eighteen local direct-versus-indirect checks were estimable; the NHFOV versus HFNC comparison for treatment failure or intubation showed an exploratory discrepancy (unadjusted *P* = 0.042). The ROP network contained no closed loops, and several other checks were not estimable because independent indirect evidence was unavailable or the direct comparison contained no events in either arm (Supplementary Tables A2, A3).

Excluding ENTARES left eight trials involving 1,202 infants for treatment failure or intubation. NIPPV and NHFOV remained associated with lower risks than NCPAP, with RRs of 0.47 (95% CI 0.34 to 0.65) and 0.62 (95% CI 0.42 to 0.90), respectively. However, the NIPPV versus HFNC comparison was no longer statistically significant (RR = 0.97, 95% CI 0.39 to 2.41). This attenuation indicates that the NIPPV versus HFNC finding was sensitive to inclusion of the trial evaluating support after stabilization, although it does not establish an interaction by treatment setting (Supplementary Table A4).

##### Publication bias

The revised comparison-adjusted funnel plots were examined descriptively because each outcome included fewer than 10 independent trials. Neither apparent symmetry nor asymmetry reliably excluded or confirmed publication bias. Egger test results were not used to draw conclusions about publication bias or the robustness of the findings.

## Discussion

This network meta-analysis of nine RCTs involving 1,449 preterm infants yielded three main findings. First, NIPPV and NHFOV were associated with lower risks of treatment failure or intubation than NCPAP, and NIPPV was also associated with a lower risk than HFNC. Second, no statistically significant differences in BPD or mortality were demonstrated among the four modes evaluated for these outcomes. Third, NIPPV was associated with a lower risk of NEC than NCPAP, although differences in severity thresholds warrant an exploratory interpretation. No significant differences were detected in the remaining safety outcomes, including IVH. These findings suggest potential benefits for avoiding treatment escalation, but do not establish corresponding reductions in major neonatal morbidity or mortality. Their interpretation depends on the populations, treatment settings, and outcome definitions represented in the network.

The respiratory findings are consistent with evidence that additional ventilatory assistance may improve the effectiveness of noninvasive support. The Cochrane review by Lemyre et al. (26) found that early NIPPV probably reduces respiratory failure and intubation compared with NCPAP, although most participants were born at approximately 28 to 32 weeks. The broader 2025 Cochrane network meta-analysis by Mukerji et al. (11) also suggested benefits of NIPPV and noninvasive high-frequency ventilation for treatment failure and endotracheal ventilation, with predominantly low or very low certainty. Agreement in the direction of these findings supports further consideration of these modes, but does not demonstrate a uniform effect across all preterm infants. These reviews also share some underlying trials, so their concordance does not constitute independent replication. The present estimates should therefore be interpreted within the clinical circumstances in which the interventions were tested.

The physiological differences between modes offer plausible explanations for respiratory benefit, but the analysis cannot separate the effects of the mode from those of its delivery. NCPAP primarily maintains distending pressure, whereas NIPPV adds intermittent inflations and NHFOV superimposes oscillations that may facilitate CO₂ clearance. The NHFOV estimate incorporates the multicenter trial by Li et al. (22), which found fewer intubations within 72 h among infants born at 24 weeks to 28 weeks and 6 days. However, the latest European consensus guidelines (27) emphasize that comparisons between modes have often involved unequal mean airway pressures, leaving uncertainty about the contribution of pressure itself. Similarly, synchronization, inspiratory time, ventilator rate, and interface leakage can influence the assistance delivered during NIPPV. Grouping synchronized and nonsynchronized NIPPV within one node therefore estimates an average effect across different implementations. This grouping was not directly tested by separating the nodes. Exclusion of individual trials cannot isolate the effect of synchronization because other study characteristics also differ. It does not establish which implementation provides the greatest benefit or whether comparable results would be obtained with different pressure settings.

The limited evidence for NIV-NAVA illustrates the distinction between physiological performance and clinical effectiveness. Its only contributing trial enrolled 20 infants and primarily evaluated respiratory effort during the first two hours after birth, leaving considerable uncertainty about treatment failure and subsequent clinical outcomes. Lee et al. (21) reported fewer neural apneic episodes without a reduction in the primary measure of respiratory effort. In a broader review, Minamitani et al. (10) found a possible reduction in treatment failure after extubation, based on two trials involving 96 infants and low-certainty evidence, but no clear benefit during primary support. This pattern does not establish an interaction by treatment setting, but it cautions against transferring post-extubation estimates to early respiratory support. Improved synchronization remains a physiological rationale for NIV-NAVA; its relative clinical value requires adequately powered comparisons within clearly defined settings.

The interpretation of treatment failure also depends on what happens after the assigned mode becomes insufficient. In the Cochrane review by Hodgson et al. (28), HFNC increased treatment failure compared with NCPAP without clearly increasing intubation, and some trials allowed rescue NCPAP before invasive ventilation. Treatment failure can therefore identify a need to change support without necessarily indicating that invasive ventilation cannot be avoided. The present analysis combined escalation to another noninvasive mode with intubation across different assessment periods. Moreover, ENTARES (17) enrolled infants after at least 48 h of stability and assessed escalation to NIPPV or mechanical ventilation, rather than exclusively early intubation. These differences may help explain why the present HFNC comparison did not reproduce the higher treatment-failure risk reported in the larger Cochrane review. The available data cannot determine their separate contributions, and the nonsignificant HFNC estimate should not be interpreted as evidence of equivalent efficacy during primary support.

The absence of a demonstrated BPD or survival benefit requires a different interpretation from the short-term respiratory findings. BPD reflects disrupted lung development and the cumulative effects of oxygen exposure, mechanical injury, inflammation, nutrition, and vascular abnormalities, as described by Ambalavanan et al. (29). Avoiding an early episode of intubation may modify part of this process, but does not necessarily establish a sustained reduction in invasive ventilation exposure or other pulmonary insults. Subsequent rescue treatment may also narrow differences between initially assigned strategies. In addition, the included trials used different BPD definitions and mortality follow-up periods. Although the Lemyre review suggested a possible reduction in chronic lung disease with NIPPV, the present estimate remained compatible with a clinically relevant benefit (26). The results are therefore not necessarily contradictory. Differences in precision and outcome classification can produce different significance thresholds even when estimates point in the same direction. For BPD and mortality, the evidence remains insufficient to determine the magnitude of any benefit.

The NEC association deserves attention because its interpretation differs from that of the respiratory outcomes. NIPPV was associated with a lower risk than NCPAP, but the upper CI was close to the null value, and NEC was one of several outcomes examined across multiple comparisons. The finding is consequently vulnerable to chance and differences in outcome classification. In particular, combining NEC reported at different severity thresholds does not produce an estimate that can be assigned confidently to a single clinically defined endpoint, such as NEC of Bell stage II or higher. The analysis also cannot determine whether any association is mediated by respiratory stability, subsequent treatment, or another component of care. An intestinal protective mechanism would therefore exceed the available evidence. The appropriate interpretation is a potentially important association that requires confirmation using consistent diagnostic criteria and sufficiently complete event reporting.

The IVH findings are constrained by both sparse events and uncertain clinical comparability. Direct HFNC versus NCPAP evidence came from three trials, with 6 events among 198 infants receiving HFNC and 1 among 196 receiving NCPAP. These trials did not report a uniform severity category: the available events included mild, severe, and insufficiently specified IVH. The pooled estimate therefore cannot be interpreted as a specific effect on severe intracranial bleeding. Timing further limits causal interpretation. Nagy et al. (30) found that the temporal distribution of IVH differed across study periods, highlighting the importance of serial imaging when evaluating exposure and event onset. ENTARES explicitly recorded comorbidities occurring after randomization, so IVH cannot uniformly be assumed to have preceded treatment (17). Nevertheless, incomplete information on baseline imaging, surveillance schedules, and individual onset times across trials prevents reliable attribution to the assigned mode. The current evidence neither establishes an HFNC-related increase in IVH nor excludes a clinically important difference, and it does not support a causal explanation based on pressure instability.

The network also requires caution because clinical differences across trials may influence indirect comparisons. Randomization protects comparisons within individual trials, but does not ensure that effect modifiers are similarly distributed across the network. Gestational age, previous ventilation, respiratory severity, treatment stage, and escalation criteria are particularly relevant in this setting. Eight trials evaluated early support, whereas ENTARES studied infants who had already achieved a period of stability. In addition, overall trial populations were analyzed without restriction to a separately reported subgroup born before 32 weeks. The analysis therefore addresses a broader population than originally specified and cannot resolve the evidence gap in extremely preterm infants. This gap also remained evident in the larger Cochrane network meta-analysis (11). The small number of trials and their uneven distribution across comparisons limit the ability to determine whether relative effects vary by maturity or clinical indication.

Statistical connectivity should likewise be distinguished from independent replication. Several connections between modes other than NCPAP were supplied by a single four-arm trial, so the number of visible edges overstates the number of independent studies supporting those comparisons. The ROP network contained no closed loops, precluding assessment of agreement between direct and indirect evidence. Rankings must be interpreted within these constraints. League-table estimates describe relative treatment effects, whereas SUCRA summarizes ranking probabilities derived from the network. A favorable ranking can coexist with a CI that includes no effect, as illustrated by NHFOV in the NEC analysis. Such a ranking does not establish superiority or compensate for sparse evidence. Clinical interpretation should therefore prioritize the estimated effect, its uncertainty, and the relevance of the contributing studies.

These limitations also affect certainty of evidence. Nonblinding does not carry the same implications for every outcome: knowledge of treatment allocation may influence discretionary escalation or intubation decisions, whereas mortality is less susceptible to ascertainment bias from nonblinding alone. Study-level risk-of-bias judgments nevertheless address only part of the uncertainty. The CINeMA framework additionally considers reporting bias, indirectness, imprecision, heterogeneity, and incoherence for each comparison and outcome. The accompanying profiles remain provisional, and overall ratings were left unassigned where outcome definitions did not permit a sufficiently coherent interpretation. These incomplete assessments limit the strength of conclusions even when individual CIs exclude the null value. Publication bias also remains unresolved: each outcome included fewer than 10 independent trials, limiting reliable assessment of small-study effects. The absence of a formal assessment should not be interpreted as evidence that publication bias was absent. Restriction to English-language full reports may have introduced further selection limitations.

The clinical relevance of this analysis lies in identifying potential advantages for avoiding treatment escalation while defining the limits of those advantages. The findings are compatible with the European consensus approach of selecting support according to gestational age, clinical condition, and the capacity to escalate treatment, rather than assuming that one mode is suitable throughout the neonatal course. NIPPV and NHFOV warrant consideration within those circumstances, but the present network does not establish a universally preferred strategy. HFNC may offer practical advantages in selected settings, yet the absence of a significant safety difference does not establish its suitability for every infant, particularly those born before 32 weeks. Conversely, the IVH findings do not justify additional HFNC-specific cranial ultrasound surveillance or recommendations to manipulate mouth closure. Such practices require evidence beyond the comparisons available here.

Future research should prioritize comparisons that distinguish the respiratory mode from the way it is delivered. A multicenter trial should enroll spontaneously breathing infants born before 32 weeks who require primary noninvasive support, with randomization stratified by center and gestational age and adequate representation of infants born before 32 weeks. Trials should report achieved pressures, synchronization, interfaces, surfactant use, and rescue pathways, and should evaluate early support, post-extubation support, and transition after stabilization separately. Using a control intubation risk of approximately 28%, as observed in the included NHFOV trial (22), detecting a reduction to 20% would require approximately 1,260 infants with equal allocation, 90% power, a two-sided *α* of 0.05, and allowance for 5% missing outcomes. This illustrative calculation should be refined using contemporary baseline risks and an agreed clinically important difference. Treatment failure and intubation should be reported as distinct outcomes within 72 h, alongside subsequent invasive ventilation exposure. Other key outcomes should include BPD at 36 weeks’ PMA, mortality before discharge, NEC of Bell stage II or higher, grade III or IV IVH with standardized imaging schedules, and pneumothorax. Longer follow-up for survival, respiratory morbidity, and neurodevelopment at 18 to 24 months’ corrected age will be necessary to determine whether short-term respiratory benefits produce lasting improvements.

## Conclusions

NIPPV and NHFOV were associated with lower risks of treatment failure or intubation than NCPAP in preterm infants, with NIPPV also showing benefit relative to HFNC. No statistically significant differences in BPD, mortality, or IVH were demonstrated. The association between NIPPV and lower NEC risk remains exploratory and requires confirmation.

Sparse evidence and differences in clinical settings and outcome definitions limit these conclusions and preclude definitive recommendations for a preferred mode. Larger multicenter RCTs with standardized outcomes are needed, particularly in infants born before 32 weeks. Clinical decisions should take into account individual patient conditions, available equipment, and team expertise.

The study used aggregate data from published trials. The extracted dataset and analysis code supporting the findings are available from the corresponding author upon request, without undue restriction.

## Data Availability

The original contributions presented in the study are included in the article/Supplementary Material, further inquiries can be directed to the corresponding authors.
