# Supplementary material for: Comparative efficacy and safety of noninvasive respiratory support in very preterm infants (<32 weeks' gestation): a network meta-analysis
Source: Front Pediatr. 2026 Oct 28;14:1928134. doi: 10.3389/fped.2026.1928134 (PMC13752198; doi:10.3389/fped.2026.1928134)
Supplement: Supplementary file 1 [file Datasheet1.zip › Supplementary material/Supplementary Table. Study-level IVH data and comparative estimates for HFNC versus NCPAP.docx]

Supplementary Table. Study-level IVH data and comparative estimates for HFNC versus NCPAP

## A. IVH definitions, ascertainment and arm-level data

| **Study** | **Events/participants** | **Definition of extracted outcome** | **Ascertainment and timing** |
| --- | --- | --- | --- |
| Gharehbaghi 2019 (16) | NIPPV: 3/30 NCPAP: 0/31 | All reported events were grade I or II. | Cranial ultrasound on days 5 to 7. Pre-existing and incident IVH were not explicitly separated. |
| Dursun 2019 (17) | NIPPV: 6/42 NCPAP: 4/42 | IVH without a separate severe-IVH count. | Ultrasound schedule and separation of pre-existing from incident events: not reported. |
| Öktem 2021 (18) | HFNC: 2/20 NCPAP: 0/20 NIPPV: 4/19 NHFOV: 0/17 | All reported events were grade I. | Ultrasound schedule and separation of pre-existing from incident events: not reported. |
| Cresi 2023 (19) | HFNC: 2/125 NCPAP: 0/122 | Severe IVH, grade >II. | Comorbidities were specified as occurring after randomization. Ultrasound schedule and individual onset dates: not reported. |
| Demirel 2021 (20) | HFNC: 2/53 NCPAP: 1/54 | IVH; severity threshold not reported. | Ultrasound schedule and separation of pre-existing from incident events: not reported. |
| Oncel 2016 (21) | NIPPV: 4/100 NCPAP: 7/100 | Severe IVH, Papile grade >II. | Ultrasound schedule and separation of pre-existing from incident events: not reported. |
| Zhang 2026 (22) | NIPPV: 4/159 NCPAP: 3/153 | Severe IVH, Papile grades III and IV. | Ultrasound schedule and separation of pre-existing from incident events: not reported. |
| Li 2025 (24) | NHFOV: 15/170 NCPAP: 16/172 | Intracranial hemorrhage grade ≥III, as reported. | Ultrasound schedule and separation of pre-existing from incident events: not reported. |

Values are events/participants in the reported outcome analysis. Trial counts overlap because some trials contributed multiple arms. Totals combine different severity categories and are not estimates of severe-IVH incidence. Lee 2022 (23) did not contribute IVH data.

“Not reported” refers to information not explicitly provided in the supplied full-text report; it does not imply that the assessment was not performed.

Supplementary Table Sx (continued)

## B. Direct HFNC versus NCPAP comparisons and network estimate

| **Study / IVH category** | **HFNC events/n** | **NCPAP events/n** | **RR (95% CI)ᵃ** | **Risk difference, percentage points (95% CI)ᵇ** |
| --- | --- | --- | --- | --- |
| Öktem 2021 (18) Grade I | 2/20 | 0/20 | 5.00 (0.26 to 98.00) | 10.00 (−7.65 to 30.10) |
| Cresi 2023 (19) Grade >II | 2/125 | 0/122 | 4.88 (0.24 to 100.64) | 1.60 (−1.67 to 5.65) |
| Demirel 2021 (20) Severity unspecified | 2/53 | 1/54 | 2.04 (0.19 to 21.81) | 1.92 (−6.46 to 11.03) |

Comparison direction: HFNC relative to NCPAP. RR >1 or a positive risk difference indicates more events with HFNC. These comparisons do not share a uniform IVH severity definition.

| **Analysis** | **Evidence contributing** | **RR (95% CI)** | **Interpretation** |
| --- | --- | --- | --- |
| Exploratory direct meta-analysisᶜ | 3 trials; 394 participants HFNC: 6/198 NCPAP: 1/196 | 3.33 (0.69 to 16.19) | Mixed IVH definitions; no statistically significant difference. |
| Supplied revised network estimateᵈ | 8 trials; 1,429 participants across 4 intervention nodes | 1.67 (0.41 to 6.67) | Mixed IVH definitions; no statistically significant difference. |

**Methods and interpretation**

ᵃ Trial-specific RRs and log-scale Wald confidence intervals were calculated from the reported arm counts. For trials with a zero-event arm, 0.5 was added to all four cells of the 2 × 2 table. Estimates are sensitive to this correction when events are sparse.

ᵇ Risk difference = HFNC risk minus NCPAP risk, expressed in percentage points. Confidence intervals use the Newcombe method based on separate Wilson score intervals, without a continuity correction.

ᶜ The direct estimate was calculated using an inverse-variance random-effects model for log RRs, with restricted maximum likelihood estimation (τ² = 0) and a normal-based 95% CI. It is an exploratory summary of the supplied mixed-definition data, not a pooled estimate of severe IVH. A common absolute risk difference was not pooled because the event definitions differed.

ᵈ The approximate network estimate is the reciprocal of the rounded supplied estimate for NCPAP versus HFNC: RR 0.60 (95% CI 0.15 to 2.44). It is not a newly fitted or severity-harmonized network estimate. Network participant totals include all four interventions, not only HFNC and NCPAP.

NNH: Only Cresi 2023 explicitly reported severe IVH in the direct HFNC comparison. Its risk difference was 1.60 percentage points (95% CI −1.67 to 5.65). The point estimate corresponds to an NNH of approximately 63, but the interval crosses zero and includes both benefit and harm; no finite two-sided NNH interval or definitive harm estimate is supported.

Abbreviations: CI, confidence interval; HFNC, high-flow nasal cannula; IVH, intraventricular hemorrhage; NCPAP, nasal continuous positive airway pressure; NHFOV, nasal high-frequency oscillatory ventilation; NIPPV, nasal intermittent positive pressure ventilation; NNH, number needed to harm; RR, risk ratio.
