# Supplementary material for: Comparative efficacy and safety of noninvasive respiratory support in very preterm infants (<32 weeks' gestation): a network meta-analysis
Source: Front Pediatr. 2026 Oct 28;14:1928134. doi: 10.3389/fped.2026.1928134 (PMC13752198; doi:10.3389/fped.2026.1928134)
Supplement: Supplementary file 1 [file Datasheet1.zip › Supplementary material/Supplementary Table. Study-specific outcome definitions and assessment periods.docx]

Supplementary Table. Study-specific outcome definitions and assessment periods

Panel A. Respiratory outcomes, BPD and mortality

| **Study** | **Respiratory failure or intubation** | **BPD** | **Mortality** |
| --- | --- | --- | --- |
| Gharehbaghi 2019 | NIV failure requiring intubation within 72 hours of admission, according to oxygenation, blood gas, apnea and clinical criteria | Supplemental oxygen or mechanical respiratory support through postnatal day 28 | Reported; endpoint timing not explicitly specified |
| Dursun 2019 | Intubation and invasive ventilation within 72 hours after birth, according to apnea, blood gas and oxygenation criteria | NIH consensus definition; severity threshold for the reported total not specified | Reported; endpoint timing not explicitly specified |
| Oktem 2021 | Intubation during noninvasive respiratory support; criteria included acidosis with hypercarbia, apnea and NEC | Reported; diagnostic criteria and assessment age not specified | Reported; endpoint timing not explicitly specified |
| Cresi 2023, ENTARES | Escalation to mechanical ventilation or NIPPV after randomization | Oxygen requirement at 36 weeks’ PMA or 28 days of life | Death before achievement of full enteral feeding |
| Demirel 2021 | Primary support failure followed by intubation; criteria included increased work of breathing, apnea, oxygen requirement and abnormal blood gases | Reported; diagnostic criteria and assessment age not specified | Reported; endpoint timing not explicitly specified |
| Oncel 2016 | Intubation within 72 hours after birth; overall intubation also reported separately | NIH criteria; moderate-to-severe BPD reported among survivors to discharge | Death reported; a separate mortality assessment window was not explicitly specified |
| Zhang 2026 | Intubation with invasive ventilation within 72 hours after birth; failure within 7 days also reported separately | Jensen 2019 criteria at 36 weeks’ PMA, with severity grades reported | In-hospital mortality |
| Lee 2022 | Intubation based on oxygen requirement, pH or apnea; allocated intervention lasted 2 hours, with subsequent clinical outcomes collected | Not reported in the supplied analysis dataset | Not reported in the supplied analysis dataset |
| Li 2025 | Invasive ventilation within 72 hours after birth; failure within 7 days also reported separately | 2019 definition; overall and moderate-to-severe BPD reported separately | In-hospital mortality |

Panel B. Safety outcomes

| **Study** | **PDA** | **ROP** | **NEC** | **IVH** | **Air leak** |
| --- | --- | --- | --- | --- | --- |
| Gharehbaghi 2019 | Diagnostic or treatment threshold not specified | Ophthalmological examination beginning 4 to 6 weeks after birth; severity threshold not specified | Stage threshold not specified | Cranial ultrasound at days 5 to 7; reported events were grades I or II, with no severe IVH | Radiographically diagnosed pneumothorax |
| Dursun 2019 | Not reported in the supplied analysis dataset | Stage ≥3 according to the Methods | Modified Bell stage ≥2 according to the Methods | Papile classification; reported total not restricted to severe IVH | Pneumothorax |
| Oktem 2021 | Reported; diagnostic threshold not specified | Not reported in the supplied analysis dataset | Reported; stage threshold not specified | Reported events were grade I | Air leak syndrome; component definition not explicitly specified |
| Cresi 2023, ENTARES | Requiring treatment | Reported; severity threshold not specified | Bell stage II or III | Severe IVH defined as grade >II | Pneumothorax |
| Demirel 2021 | Reported; diagnostic threshold not specified | Reported; severity threshold not specified | Reported; stage threshold not specified | Reported; severity threshold not specified | Pneumothorax |
| Oncel 2016 | Echocardiographic assessment at 48 to 96 hours; medication and surgical ligation reported separately | Requiring laser treatment | Modified Bell stage ≥II | Papile grade >II | Pneumothorax |
| Zhang 2026 | Hemodynamically significant PDA | Table reports stage ≥2 | Any grade according to the Methods | Papile grades III or IV | Pneumothorax |
| Lee 2022 | Not reported in the supplied analysis dataset | Not reported in the supplied analysis dataset | Not reported in the supplied analysis dataset | Not reported in the supplied analysis dataset | Pneumothorax; the reported event occurred after the allocated intervention and subsequent intubation |
| Li 2025 | Hemodynamically significant PDA specified in the Methods; outcome table labelled PDA | Stage >II | Stage ≥II | Grade ≥III | Pneumothorax or other air leaks during assigned treatment |
