# Supplementary material for: Comparative efficacy and safety of noninvasive respiratory support in very preterm infants (<32 weeks' gestation): a network meta-analysis
Source: Front Pediatr. 2026 Oct 28;14:1928134. doi: 10.3389/fped.2026.1928134 (PMC13752198; doi:10.3389/fped.2026.1928134)
Supplement: Supplementary file 1 [file Datasheet1.zip › Supplementary material/Supplementary figure1. Risk of bias assessment results.docx]

**A. Risk of bias graph.**

**B. Risk of bias summary**.

**Figure1. Risk of bias assessment results of the included studies.**

"+" indicates low risk of bias; "?" indicates unclear risk of bias; "-" indicates high risk of bias.
