# Supplementary material for: Comparative efficacy and safety of noninvasive respiratory support in very preterm infants (<32 weeks' gestation): a network meta-analysis
Source: Front Pediatr. 2026 Oct 28;14:1928134. doi: 10.3389/fped.2026.1928134 (PMC13752198; doi:10.3389/fped.2026.1928134)
Supplement: Supplementary file 1 [file Datasheet1.zip › Supplementary material/Supplementary table S1. Search strategies.docx]

**Table1. Search strategies for PubMed.**

| Search number | Query |
| --- | --- |
| #1 | "Infant, Premature"[Mesh] |
| #2 | "Premature Birth"[Mesh] |
| #3 | "Infant, Extremely Premature"[Mesh] |
| #4 | (very preterm*[Title/Abstract]) OR (extremely preterm*[Title/Abstract]) OR (extremely premature*[Title/Abstract]) OR (extremely low birth weight[Title/Abstract]) OR (ELBW[Title/Abstract]) OR (very low birth weight[Title/Abstract]) OR (VLBW[Title/Abstract]) OR (preterm*[Title/Abstract]) OR (premature*[Title/Abstract]) OR (neonate*[Title/Abstract]) OR (newborn*[Title/Abstract]) |
| #5 | #1 OR #2 OR #3 OR #4 |
| #6 | "Noninvasive Ventilation"[Mesh] |
| #7 | (noninvasive ventilat*[Title/Abstract]) OR (non Invasive Ventilat*[Title/Abstract]) OR (non-Invasive Ventilat*[Title/Abstract]) OR (Mechanical Ventilat*[Title/Abstract]) OR (nrs[Title/Abstract]) |
| #8 | "Continuous Positive Airway Pressure"[Mesh] |
| #9 | (continuous positive airway* pressure*[Title/Abstract]) OR (CPAP[Title/Abstract] OR C-PAP[Title/Abstract] OR NCPAP[Title/Abstract] OR n-CPAP [Title/Abstract]) OR (positive pressure* respirat*[Title/Abstract]) OR (Positive-Pressure* Ventilat*[Title/Abstract]) OR (Positive Pressure* Ventilat*[Title/Abstract]) OR (PPR[Title/Abstract]) |
| #10 | "Intermittent Positive-Pressure Breathing"[Mesh] |
| #11 | "Intermittent Positive-Pressure Ventilation"[Mesh] |
| #12 | (Intermittent Positive Pressure Breathing [Title/Abstract]) OR (Intermittent Positive Pressure Ventilat* [Title/Abstract]) OR (IPPB[Title/Abstract]) OR (IPPV[Title/Abstract]) |
| #13 | "High-Frequency Ventilation"[Mesh] |
| #14 | (High Frequency Ventilat*[Title/Abstract]) OR (High-Frequency Oscillation Ventilat*[Title/Abstract]) OR (High Frequency Oscillation Ventilat*[Title/Abstract]) OR (NHFOV[Title/Abstract] OR NIHFOV[Title/Abstract] OR NHFV[Title/Abstract] OR NIHFV[Title/Abstract]) |
| #15 | (High-Flow Nasal Cannula*[Title/Abstract]) OR (high flow Nasal Cannula*[Title/Abstract]) OR (highflow Nasal Cannula*[Title/Abstract]) OR (High-Flow Oxygen Therapy[Title/Abstract]) OR (high flow Oxygen Therapy[Title/Abstract]) OR (highflow Oxygen Therapy[Title/Abstract]) OR (high-flow Ventilat*[Title/Abstract]) OR (high flow Ventilat*[Title/Abstract]) OR (highflow Ventilat*[Title/Abstract]) OR (high-flow respirat*[Title/Abstract]) OR (high flow respirat*[Title/Abstract]) OR (highflow respirat*[Title/Abstract]) OR (HFNC[Title/Abstract]) OR (HHHFNC[Title/Abstract]) |
| #16 | (neurally adjusted ventilat* assist*[Title/Abstract]) OR (Diaphragm-triggered non-invasive respirat* support[Title/Abstract]) OR (NIV-NAVA[Title/Abstract] OR NAVA[Title/Abstract]) |
| #17 | #6 OR #7 OR #8 OR #9 OR #10 OR #11 OR #12 OR #13 OR #14 OR #15 OR #16 |
| #18 | "Randomized Controlled Trial" [Publication Type] |
| #19 | "Randomized Controlled Trials as Topic"[Mesh] |
| #20 | (randomized[Title/Abstract]) OR (randomised[Title/Abstract]) OR (placebo[Title/Abstract]) OR (randomly[Title/Abstract]) OR (trial[Title/Abstract]) OR (groups[Title/Abstract]) |
| #21 | #18 OR #19 OR #20 |
| #22 | #5 AND #17 AND #21 |
