# Supplementary material for: Comparative efficacy and safety of noninvasive respiratory support in very preterm infants (<32 weeks' gestation): a network meta-analysis
Source: Front Pediatr. 2026 Oct 28;14:1928134. doi: 10.3389/fped.2026.1928134 (PMC13752198; doi:10.3389/fped.2026.1928134)
Supplement: Supplementary file 1 [file Datasheet1.zip › Supplementary material/Supplementary. Cinema.docx]

CINeMA outcomes

| **Outcome / scope** | **Comparison (first vs second)** | **RR (95% CI)** | **Within-study bias** | **Reporting bias** | **Indirectness** | **Imprecision** | **Heterogeneity** | **Incoherence** | **Overall confidence** |
| --- | --- | --- | --- | --- | --- | --- | --- | --- | --- |
| Failure or intubation | NIPPV vs NHFOV | 0.76 (0.46 to 1.24) | Some concerns | Suspected | Some concerns | Some concerns | No concerns | No concerns | Very low |
| Failure or intubation | NIPPV vs NIV-NAVA | 0.63 (0.18 to 2.21) | Some concerns | Suspected | Some concerns | Major concerns | No concerns | No concerns | Very low |
| Failure or intubation | NHFOV vs NIV-NAVA | 0.83 (0.23 to 2.97) | Some concerns | Suspected | Some concerns | Major concerns | No concerns | No concerns | Very low |
| Failure or intubation | NIPPV vs HFNC | 0.53 (0.29 to 0.98) | Some concerns | Suspected | Some concerns | No concerns | No concerns | No concerns | Very low |
| Failure or intubation | NHFOV vs HFNC | 0.71 (0.38 to 1.33) | Some concerns | Suspected | Some concerns | Major concerns | No concerns | Major concerns | Very low |
| Failure or intubation | NIV-NAVA vs HFNC | 0.85 (0.23 to 3.18) | Some concerns | Suspected | Some concerns | Major concerns | No concerns | No concerns | Very low |
| Failure or intubation | NIPPV vs NCPAP | 0.47 (0.34 to 0.65) | Some concerns | Suspected | No concerns | No concerns | No concerns | No concerns | Low |
| Failure or intubation | NHFOV vs NCPAP | 0.62 (0.43 to 0.91) | Some concerns | Suspected | No concerns | No concerns | No concerns | No concerns | Low |
| Failure or intubation | NIV-NAVA vs NCPAP | 0.75 (0.22 to 2.52) | Some concerns | Suspected | Some concerns | Major concerns | No concerns | No concerns | Very low |
| Failure or intubation | HFNC vs NCPAP | 0.88 (0.53 to 1.48) | Some concerns | Suspected | Major concerns | Major concerns | No concerns | No concerns | Very low |
| BPD | NHFOV vs HFNC | 0.88 (0.40 to 1.97) | Some concerns | Suspected | Major concerns | Major concerns | No concerns | No concerns | Not assessable |
| BPD | NHFOV vs NIPPV | 0.87 (0.42 to 1.76) | Some concerns | Suspected | Major concerns | Major concerns | No concerns | No concerns | Not assessable |
| BPD | HFNC vs NIPPV | 0.98 (0.50 to 1.90) | Some concerns | Suspected | Major concerns | Major concerns | No concerns | No concerns | Not assessable |
| BPD | NHFOV vs NCPAP | 0.72 (0.38 to 1.40) | No concerns | Suspected | Major concerns | Major concerns | No concerns | No concerns | Not assessable |
| BPD | HFNC vs NCPAP | 0.82 (0.46 to 1.47) | No concerns | Suspected | Major concerns | Major concerns | No concerns | No concerns | Not assessable |
| BPD | NIPPV vs NCPAP | 0.84 (0.54 to 1.29) | Some concerns | Suspected | Major concerns | Major concerns | No concerns | No concerns | Not assessable |
| Mortality | NIPPV vs NHFOV | 0.94 (0.29 to 3.01) | No concerns | Suspected | Some concerns | Major concerns | No concerns | No concerns | Very low |
| Mortality | NIPPV vs HFNC | 0.90 (0.15 to 5.57) | No concerns | Suspected | Some concerns | Major concerns | No concerns | No concerns | Very low |
| Mortality | NHFOV vs HFNC | 0.96 (0.16 to 5.83) | No concerns | Suspected | Some concerns | Major concerns | No concerns | No concerns | Very low |
| Mortality | NIPPV vs NCPAP | 0.69 (0.29 to 1.63) | Some concerns | Suspected | No concerns | Major concerns | No concerns | No concerns | Very low |
| Mortality | NHFOV vs NCPAP | 0.74 (0.33 to 1.66) | No concerns | Suspected | No concerns | Major concerns | No concerns | No concerns | Very low |
| Mortality | HFNC vs NCPAP | 0.77 (0.15 to 3.91) | No concerns | Suspected | Some concerns | Major concerns | No concerns | No concerns | Very low |
| ROP | NIPPV vs NCPAP | 0.89 (0.65 to 1.21) | Major concerns | Suspected | Major concerns | Some concerns | No concerns | Major concerns | Not assessable |
| ROP | NIPPV vs NHFOV | 0.86 (0.52 to 1.40) | Some concerns | Suspected | Major concerns | Major concerns | No concerns | Major concerns | Not assessable |
| ROP | NCPAP vs NHFOV | 0.96 (0.65 to 1.42) | No concerns | Suspected | Major concerns | Major concerns | No concerns | Major concerns | Not assessable |
| ROP | NIPPV vs HFNC | 0.74 (0.38 to 1.45) | Some concerns | Suspected | Major concerns | Major concerns | No concerns | Major concerns | Not assessable |
| ROP | NCPAP vs HFNC | 0.83 (0.46 to 1.51) | No concerns | Suspected | Major concerns | Major concerns | No concerns | Major concerns | Not assessable |
| ROP | NHFOV vs HFNC | 0.86 (0.42 to 1.76) | No concerns | Suspected | Major concerns | Major concerns | No concerns | Major concerns | Not assessable |
| PDA | HFNC vs NCPAP | 0.88 (0.55 to 1.43) | No concerns | Suspected | Major concerns | Major concerns | No concerns | No concerns | Not assessable |
| PDA | HFNC vs NIPPV | 0.87 (0.52 to 1.47) | Some concerns | Suspected | Major concerns | Major concerns | No concerns | No concerns | Not assessable |
| PDA | NCPAP vs NIPPV | 0.99 (0.81 to 1.21) | Major concerns | Suspected | Major concerns | No concerns | No concerns | No concerns | Not assessable |
| PDA | HFNC vs NHFOV | 0.84 (0.45 to 1.56) | No concerns | Suspected | Major concerns | Major concerns | No concerns | No concerns | Not assessable |
| PDA | NCPAP vs NHFOV | 0.95 (0.63 to 1.42) | No concerns | Suspected | Major concerns | Major concerns | No concerns | No concerns | Not assessable |
| PDA | NIPPV vs NHFOV | 0.96 (0.61 to 1.50) | Some concerns | Suspected | Major concerns | Major concerns | No concerns | No concerns | Not assessable |
| NEC | NHFOV vs NIPPV | 0.92 (0.42 to 2.03) | Some concerns | Suspected | Major concerns | Major concerns | No concerns | No concerns | Not assessable |
| NEC | NHFOV vs HFNC | 0.54 (0.17 to 1.77) | No concerns | Suspected | Major concerns | Major concerns | No concerns | No concerns | Not assessable |
| NEC | NIPPV vs HFNC | 0.59 (0.20 to 1.75) | Some concerns | Suspected | Major concerns | Major concerns | No concerns | No concerns | Not assessable |
| NEC | NHFOV vs NCPAP | 0.55 (0.29 to 1.04) | No concerns | Suspected | Major concerns | Some concerns | No concerns | No concerns | Not assessable |
| NEC | NIPPV vs NCPAP | 0.60 (0.37 to 0.97) | Major concerns | Suspected | Major concerns | No concerns | No concerns | No concerns | Not assessable |
| NEC | HFNC vs NCPAP | 1.02 (0.36 to 2.87) | No concerns | Suspected | Major concerns | Major concerns | No concerns | No concerns | Not assessable |
| IVH | NHFOV vs NCPAP | 0.82 (0.31 to 2.22) | No concerns | Suspected | Major concerns | Major concerns | No concerns | No concerns | Not assessable |
| IVH | NHFOV vs HFNC | 0.50 (0.10 to 2.38) | No concerns | Suspected | Major concerns | Major concerns | No concerns | No concerns | Not assessable |
| IVH | NCPAP vs HFNC | 0.60 (0.17 to 2.17) | Some concerns | Suspected | Major concerns | Major concerns | No concerns | No concerns | Not assessable |
| IVH | NHFOV vs NIPPV | 0.51 (0.15 to 1.72) | Some concerns | Suspected | Major concerns | Major concerns | No concerns | No concerns | Not assessable |
| IVH | NCPAP vs NIPPV | 0.62 (0.29 to 1.34) | Some concerns | Suspected | Major concerns | Major concerns | No concerns | No concerns | Not assessable |
| IVH | HFNC vs NIPPV | 1.04 (0.29 to 3.76) | Some concerns | Suspected | Major concerns | Major concerns | No concerns | No concerns | Not assessable |
| Air leak | HFNC vs NHFOV | 0.86 (0.10 to 7.19) | Some concerns | Suspected | Some concerns | Major concerns | No concerns | No concerns | Very low |
| Air leak | HFNC vs NCPAP | 0.61 (0.12 to 3.19) | No concerns | Suspected | No concerns | Major concerns | No concerns | No concerns | Very low |
| Air leak | NHFOV vs NCPAP | 0.71 (0.15 to 3.46) | No concerns | Suspected | No concerns | Major concerns | No concerns | No concerns | Very low |
| Air leak | HFNC vs NIPPV | 0.61 (0.10 to 3.83) | Some concerns | Suspected | Some concerns | Major concerns | No concerns | No concerns | Very low |
| Air leak | NHFOV vs NIPPV | 0.70 (0.13 to 3.76) | Some concerns | Suspected | Some concerns | Major concerns | No concerns | No concerns | Very low |
| Air leak | NCPAP vs NIPPV | 0.99 (0.36 to 2.71) | Some concerns | Suspected | No concerns | Major concerns | No concerns | No concerns | Very low |
| Air leak | HFNC vs NIV-NAVA | 0.20 (0.01 to 7.35) | Some concerns | Suspected | Some concerns | Major concerns | No concerns | No concerns | Very low |
| Air leak | NHFOV vs NIV-NAVA | 0.24 (0.01 to 8.26) | Some concerns | Suspected | Some concerns | Major concerns | No concerns | No concerns | Very low |
| Air leak | NCPAP vs NIV-NAVA | 0.33 (0.01 to 8.00) | Some concerns | Suspected | Major concerns | Major concerns | No concerns | No concerns | Very low |
| Air leak | NIPPV vs NIV-NAVA | 0.34 (0.01 to 9.44) | Some concerns | Suspected | Some concerns | Major concerns | No concerns | No concerns | Very low |
| Severe IVH (grade III or IV) | NCPAP vs HFNC | 0.20 (0.01 to 4.22) | No concerns | Suspected | Major concerns | Major concerns | No concerns | Major concerns | Very low |
| Severe IVH (grade III or IV) | NCPAP vs NHFOV | 1.05 (0.54 to 2.06) | No concerns | Suspected | No concerns | Major concerns | No concerns | Major concerns | Very low |
| Severe IVH (grade III or IV) | NCPAP vs NIPPV | 1.27 (0.50 to 3.22) | Some concerns | Suspected | No concerns | Major concerns | No concerns | Major concerns | Very low |
| Severe IVH (grade III or IV) | HFNC vs NHFOV | 5.15 (0.23 to 114.22) | No concerns | Suspected | Some concerns | Major concerns | No concerns | Major concerns | Very low |
| Severe IVH (grade III or IV) | HFNC vs NIPPV | 6.20 (0.26 to 147.14) | Some concerns | Suspected | Some concerns | Major concerns | No concerns | Major concerns | Very low |
| Severe IVH (grade III or IV) | NHFOV vs NIPPV | 1.21 (0.38 to 3.80) | Some concerns | Suspected | Some concerns | Major concerns | No concerns | Major concerns | Very low |
