# Supplementary material for: Comparative efficacy and safety of noninvasive respiratory support in very preterm infants (<32 weeks' gestation): a network meta-analysis
Source: Front Pediatr. 2026 Oct 28;14:1928134. doi: 10.3389/fped.2026.1928134 (PMC13752198; doi:10.3389/fped.2026.1928134)
Supplement: Supplementary file 1 [file Datasheet1.zip › Supplementary material/Supplementary. Statistical method and trial characteristics.docx]

Supplementary statistical method

# Supplementary analysis methods

Eight outcome networks were reconstructed from the extraction workbook. Study identities were matched by author and year; Lee 2022 and Li 2025 were normalized to study IDs 23 and 24, respectively. Event counts and denominators were unchanged. Mixed outcome definitions, including differing BPD and IVH severity thresholds, were retained for reproducibility checks and limit clinical interpretation.

The independent analysis used Python 3.12.14, NumPy 2.3.5, and SciPy 1.17.0. A contrast-based multivariate normal model was fitted to log RRs. A common between-study variance within each outcome network was estimated by restricted maximum likelihood (REML). RRs were selected to express relative event probabilities directly; no rare-event approximation from ORs was used. RR below 1 favors the first treatment.

For a trial with k arms, k−1 contrasts were modeled jointly. Sampling covariance between contrasts sharing a reference arm equaled the variance of that arm’s log risk. Random-effects variances and covariances were τ² and τ²/2, respectively. Trials were not divided into independent pairwise comparisons. Where an arm had zero events, 0.5 was added to the event and nonevent count in every arm of that trial. All-zero trials were retained in descriptive counts but omitted from the main log-RR audit fit. Zero-event handling was examined in sensitivity analyses.

Normal-approximation 95% CIs and plug-in 95% prediction intervals were calculated on the log-RR scale. Prediction variance was the variance of the estimated mean log RR plus τ². These exploratory intervals do not incorporate uncertainty in τ² and may understate predictive uncertainty in sparse networks. The total Q-based I² includes departures due to heterogeneity and incoherence; it is not a comparison-specific I².

Global incoherence was examined by the generalized least-squares Q difference between the consistency model and a saturated model of design-specific treatment effects, conditional on the consistency model’s estimated τ². Local checks compared direct evidence with an indirect network excluding all trials that directly compared the target treatments. The same τ² was used on both sides. Double-zero direct contrasts were not estimated. Tests were two-sided, unadjusted, and exploratory; a nonsignificant result does not establish coherence.

Post hoc sensitivity analyses examined a common-effect model, exclusion of ENTARES, exclusion of Zhang 2026, exclusion of the four-arm Öktem trial, continuity corrections of 0.1 and 1.0, and retention of all-zero trials using a 0.5 correction. Clinical-setting analyses were not described as prespecified. No separate postextubation network was available.

# Table A1 Trial characteristics relevant to transitivity

Panel A. Populations and recruitment periods. Values separated by slashes follow the arm order shown. Central values are displayed for compact cross-trial comparison; mean and median values have not been pooled or converted.

| **Study and setting** | **Arms and sample sizes** | **GA eligibility (wk)** | **Arm GA (wk)** | **Arm BW (g)** |
| --- | --- | --- | --- | --- |
| Gharehbaghi 2019 Iran; Jan to Sep 2017 | NIPPV 30 / NCPAP 31 | 28 to 32 | 30.4 / 29.8 (mean) | 1301 / 1246 (mean) |
| Dursun 2019 Turkey; Nov 2013 to Nov 2014 | NIPPV 42 / NCPAP 42 | 24 to 32 | 29.2 / 29.4 (mean) | 1356 / 1359 (mean) |
| Öktem 2021 Turkey; Dec 2015 to Feb 2017 | HFNC 20 / NCPAP 20 / NIPPV 19 / NHFOV 17 | <32 | 28 / 28 / 28 / 29 (median) | 1190 / 1240 / 1130 / 1250 (median) |
| Cresi 2023 Italy; Nov 2018 to Jun 2021 | NCPAP 122 / HFNC 125 | 25 to 29 | 28 / 28 (median) | 1054 / 1085 (mean) |
| Demirel 2021 Turkey; Feb 2017 to Feb 2018 | HFNC 53 / NCPAP 54 | ≤32 | 31.2 / 31.0 (median) | 1570 / 1505 (median) |
| Oncel 2016 Turkey; Nov 2012 to Nov 2013 | NCPAP 100 / NIPPV 100 | 26 to 32 | 29.1 / 29.2 (mean) | 1175 / 1180 (mean) |
| Zhang 2026 China; Dec 2021 to Oct 2024 | NCPAP 153 / NIPPV 159 | 24 to 29+6 | 28.5 / 29.0 (median) | 1120 / 1130 (median) |
| Lee 2022 Finland; Jan 2017 to Jul 2019 | NIV-NAVA 10 / NCPAP 10 | 28+0 to 31+6 | 29+4 / 29+6 (mean; weeks+days) | 1331 / 1346 (mean) |
| Li 2025 China; Aug 2022 to Aug 2024 | NHFOV 170 / NCPAP 172 | 24 to 28+6 | 27 / 27 (median) | 990 / 970 (median) |

All nine studies contributed whole-trial data. No verified GA <28-week subgroup was extracted. Eight trials evaluated early support; ENTARES evaluated support after clinical stabilization. Lee 2022 was conducted in Finland, irrespective of author affiliations elsewhere.

# Table A1 Continued

Panel B. Antenatal corticosteroids, clinical setting, and surfactant strategy. Arm order is the same as Panel A. Surfactant given after randomization is a cointervention or outcome, not a baseline characteristic.

| **Study** | **Antenatal corticosteroids** | **Clinical setting and surfactant strategy** |
| --- | --- | --- |
| Gharehbaghi 2019 | None / one dose / two doses: NIPPV 1 / 19 / 9; NCPAP 2 / 11 / 18. One NIPPV participant is not accounted for by these reported categories. | Early support. No prophylactic surfactant; rescue poractant alfa by INSURE. Surfactant during follow-up: 15/30 vs 19/31. Synchronization not clearly specified. |
| Dursun 2019 | Any exposure: 20/42 (47.6%) vs 22/42 (52.4%), calculated by summing the reported one-course and two-course categories. | Early support; synchronized NIPPV. Intubation and surfactant after support failure: 5/42 vs 17/42. Prior intubation or surfactant within the first hour was an exclusion criterion. |
| Öktem 2021 | 6/20 (30%) / 5/20 (25%) / 7/19 (37%) / 11/17 (65%). | Early support; nonsynchronized NIPPV. Rescue poractant alfa for FiO₂ >0.40; dose categories reported separately. Four arms belong to one trial. |
| Cresi 2023 | 112/122 (91.8%) vs 115/125 (92.0%); steroid prophylaxis as reported. | Stable on NRS for ≥48 h; median recruitment age 4 vs 5 d. Surfactant before recruitment: 64/122 (52.5%) vs 71/125 (56.8%). Previous postextubation support permitted. |
| Demirel 2021 | 38/53 (71.6%) vs 40/54 (74.0%). | Primary support. Rescue poractant alfa by INSURE for radiographic RDS and FiO₂ >0.40. One-dose category: 13 vs 21; two-dose category: 5 vs 3. |
| Oncel 2016 | 85/100 (85%) vs 86/100 (86%). | Early support; nonsynchronized NIPPV. MIST after randomization when indicated. Surfactant during follow-up: 60/100 vs 38/100. |
| Zhang 2026 | Any exposure: 126/153 (82.4%) vs 132/159 (83.0%); full course: 93/153 (60.8%) vs 98/159 (61.6%). | Early support; synchronized NIPPV. MISA within 120 min after birth per protocol. More than one dose: 31/153 vs 32/159. Trial stopped early. |
| Lee 2022 | Full prenatal course: 9/10 (90%) in each arm. | Assigned support immediately after birth for 2 h. No arm-level baseline surfactant exposure tabulated. Subsequent intubation and surfactant occurred during follow-up. |
| Li 2025 | 123/170 (72.4%) vs 121/172 (70.3%); prenatal prophylaxis as reported. | Primary support. Rescue poractant alfa using LISA according to oxygen requirement; repeat doses permitted. Arm-level baseline surfactant exposure not tabulated. |

Steroid definitions differ across trials. The Gharehbaghi dose-category discrepancy is reproduced explicitly and has not been imputed. Counts for surfactant during follow-up must not be used as baseline adjustment variables. INSURE, intubation, surfactant, extubation; MIST, minimally invasive surfactant therapy; MISA, minimally invasive surfactant administration; LISA, less invasive surfactant administration; NRS, noninvasive respiratory support.

# Table A2 Network heterogeneity and global incoherence

| **Outcome** | **Trials reported / modeled** | **Infants reported / modeled** | **τ²** | **Total I² (%)** | **Global Q / df / P** |
| --- | --- | --- | --- | --- | --- |
| Failure or intubation | 9 / 9 | 1449 / 1449 | 0.0000 | 38.0 | 4.881 / 3 / 0.181 |
| BPD | 8 / 8 | 1429 / 1429 | 0.1094 | 46.5 | 5.967 / 3 / 0.113 |
| Mortality | 8 / 7 | 1429 / 1322 | 0.0000 | 0.0 | 0.486 / 3 / 0.922 |
| ROP | 7 / 7 | 1353 / 1353 | 0.0000 | 0.0 | Not estimable |
| PDA | 7 / 7 | 1345 / 1345 | 0.0000 | 0.0 | 0.936 / 3 / 0.817 |
| NEC | 8 / 8 | 1429 / 1429 | 0.0000 | 0.0 | 1.611 / 3 / 0.657 |
| IVH | 8 / 8 | 1429 / 1429 | 0.1700 | 2.5 | 3.121 / 3 / 0.373 |
| Air leak | 9 / 9 | 1449 / 1449 | 0.1450 | 0.0 | 1.999 / 3 / 0.573 |

These diagnostics belong exclusively to the independent REML audit. Global tests are conditional on the estimated τ², not a recovered Stata design-by-treatment output. All-zero mortality events in Demirel 2021 explain the difference between reported and modeled sample sizes. ROP has no independently informative closed loops, so its global test has no degrees of freedom.

A zero τ² estimate does not demonstrate clinical homogeneity. Total I² is calculated from the common-effect residual Q and may differ in behavior from REML τ². In particular, it does not separate within-design heterogeneity from incoherence.

# Zero-event handling

| **Outcome** | **Trials receiving continuity correction** | **All-zero trials omitted from main audit** |
| --- | --- | --- |
| Failure or intubation | None | None |
| BPD | None | None |
| Mortality | Gharehbaghi 2019, Öktem 2021 | Demirel 2021 |
| ROP | None | None |
| PDA | None | None |
| NEC | Gharehbaghi 2019, Öktem 2021 | None |
| IVH | Gharehbaghi 2019, Öktem 2021, Cresi 2023 | None |
| Air leak | Gharehbaghi 2019, Öktem 2021, Cresi 2023, Lee 2022 | None |

# Table A3 Local direct and indirect evidence checks

| **Outcome** | **Comparison** | **Direct RR (95% CI)** | **Indirect RR (95% CI)** | **P** |
| --- | --- | --- | --- | --- |
| Failure or intubation | NIPPV vs NHFOV | 0.36 (0.08 to 1.61) | 0.85 (0.50 to 1.46) | 0.286 |
| Failure or intubation | NIPPV vs HFNC | 1.05 (0.16 to 6.74) | 0.45 (0.23 to 0.86) | 0.395 |
| Failure or intubation | NHFOV vs HFNC | 2.94 (0.65 to 13.27) | 0.52 (0.26 to 1.06) | 0.042 |
| BPD | NIPPV vs NHFOV | 1.49 (0.54 to 4.09) | 1.06 (0.38 to 2.93) | 0.639 |
| BPD | NIPPV vs HFNC | 1.75 (0.63 to 4.89) | 0.71 (0.30 to 1.69) | 0.186 |
| BPD | NHFOV vs HFNC | 1.18 (0.38 to 3.65) | 0.67 (0.21 to 2.10) | 0.493 |
| PDA | NIPPV vs NHFOV | 1.49 (0.42 to 5.33) | 0.90 (0.56 to 1.46) | 0.469 |
| PDA | NIPPV vs HFNC | 1.75 (0.48 to 6.35) | 1.05 (0.60 to 1.87) | 0.479 |
| PDA | NHFOV vs HFNC | 1.18 (0.27 to 5.09) | 1.17 (0.59 to 2.32) | 0.994 |
| NEC | NIPPV vs NHFOV | 2.70 (0.12 to 62.17) | 1.01 (0.45 to 2.29) | 0.554 |
| NEC | NIPPV vs HFNC | 0.63 (0.09 to 4.34) | 0.74 (0.18 to 2.96) | 0.899 |
| NEC | NHFOV vs HFNC | 0.23 (0.01 to 4.55) | 0.73 (0.17 to 3.10) | 0.501 |
| IVH | NIPPV vs NHFOV | 8.10 (0.42 to 156.91) | 1.24 (0.32 to 4.75) | 0.258 |
| IVH | NIPPV vs HFNC | 1.89 (0.37 to 9.68) | 0.41 (0.05 to 3.45) | 0.265 |
| IVH | NHFOV vs HFNC | 0.23 (0.01 to 5.07) | 0.33 (0.04 to 3.05) | 0.857 |
| Air leak | NIPPV vs NHFOV | 0.90 (0.09 to 8.94) | 3.89 (0.30 to 50.28) | 0.404 |
| Air leak | NIPPV vs HFNC | 3.15 (0.12 to 79.54) | 1.20 (0.13 to 11.27) | 0.631 |
| Air leak | NHFOV vs HFNC | 3.50 (0.14 to 88.09) | 0.31 (0.02 to 6.30) | 0.281 |

Each displayed direct estimate comes from Öktem 2021; indirect evidence comes from other trials. All 56 comparisons, including non-estimable checks and contributing trial IDs, are documented in local_checks.csv. These conservative checks are not an exhaustive decomposition of the global design-by-treatment discrepancy. Geometric loops are not counted as separate replications.

ROP has no closed loops. In the other networks, removing all directly comparing trials leaves no independent indirect path for NCPAP contrasts; NIV-NAVA is attached by one trial only. For mortality, the three peripheral direct comparisons in Öktem are double-zero and are not estimated. Thus a graphically closed loop does not guarantee an estimable local test.

For treatment failure or intubation, NHFOV versus HFNC showed discordant direct and indirect estimates (unadjusted P = 0.042). This exploratory result is sensitive to sparse data and multiplicity, but it prevents a blanket statement that all evidence sources were consistent.

# Table A4 Post hoc sensitivity analyses

| **Outcome and comparison** | **All trials in audit** | **Exclude ENTARES** | **Exclude Zhang** | **Exclude Öktem** |
| --- | --- | --- | --- | --- |
| Failure or intubation NIPPV vs NCPAP | 0.47 (0.34 to 0.65) | 0.47 (0.34 to 0.65) | 0.47 (0.25 to 0.87) | 0.49 (0.35 to 0.68) |
| Failure or intubation NHFOV vs NCPAP | 0.62 (0.43 to 0.91) | 0.62 (0.42 to 0.90) | 0.71 (0.36 to 1.38) | 0.57 (0.37 to 0.87) |
| Failure or intubation NIPPV vs HFNC | 0.53 (0.29 to 0.98) | 0.97 (0.39 to 2.41) | 0.60 (0.24 to 1.52) | 0.45 (0.23 to 0.86) |
| NEC NIPPV vs NCPAP | 0.60 (0.37 to 0.97) | 0.61 (0.37 to 0.99) | 0.64 (0.24 to 1.66) | 0.58 (0.35 to 0.95) |

All cells show RR (95% CI), estimated afresh under the same REML model. The ENTARES exclusion leaves eight trials and 1,202 infants for treatment failure or intubation. NIPPV versus HFNC changes from 0.53 (0.29 to 0.98) to 0.97 (0.39 to 2.41). NIPPV and NHFOV versus NCPAP retain lower estimated risks, with 95% CIs excluding 1 in that particular sensitivity analysis.

ENTARES alone reported 18/125 events with HFNC and 14/122 with NCPAP, giving RR 1.25 (95% CI 0.65 to 2.41). This outcome included escalation to mechanical ventilation or NIPPV. ENTARES cannot be relabeled as a pure postextubation trial, and its single comparison cannot support a separate five-mode network or a general treatment-by-setting interaction.

Exclusion of Zhang 2026 widened uncertainty, including for NHFOV versus NCPAP, despite Zhang not directly studying NHFOV. This illustrates the dependence of sparse-network estimates on a shared heterogeneity parameter. Changes in statistical significance are not tests of treatment-effect interaction.

All 56 comparisons under all seven sensitivity specifications are provided in sensitivity_results.csv. These analyses were undertaken during revision and are post hoc. The numerical audit does not resolve the mixed definitions of BPD, NEC, IVH, and other outcomes.

# Table A5 Prediction intervals for comparisons with NCPAP

| **Outcome** | **Comparison** | **RR (95% CI)** | **95% prediction interval** |
| --- | --- | --- | --- |
| Failure or intubation | NIPPV vs NCPAP | 0.47 (0.34 to 0.65) | 0.34 to 0.65 |
| Failure or intubation | NHFOV vs NCPAP | 0.62 (0.43 to 0.91) | 0.43 to 0.91 |
| Failure or intubation | HFNC vs NCPAP | 0.88 (0.53 to 1.48) | 0.53 to 1.48 |
| Failure or intubation | NIV-NAVA vs NCPAP | 0.75 (0.22 to 2.52) | 0.22 to 2.52 |
| BPD | NIPPV vs NCPAP | 0.84 (0.54 to 1.29) | 0.38 to 1.83 |
| BPD | NHFOV vs NCPAP | 0.72 (0.38 to 1.40) | 0.29 to 1.82 |
| BPD | HFNC vs NCPAP | 0.82 (0.46 to 1.47) | 0.34 to 1.96 |
| Mortality | NIPPV vs NCPAP | 0.69 (0.29 to 1.63) | 0.29 to 1.63 |
| Mortality | NHFOV vs NCPAP | 0.74 (0.33 to 1.66) | 0.33 to 1.66 |
| Mortality | HFNC vs NCPAP | 0.77 (0.15 to 3.91) | 0.15 to 3.91 |
| ROP | NIPPV vs NCPAP | 0.89 (0.65 to 1.21) | 0.65 to 1.21 |
| ROP | NHFOV vs NCPAP | 1.04 (0.70 to 1.53) | 0.70 to 1.53 |
| ROP | HFNC vs NCPAP | 1.20 (0.66 to 2.18) | 0.66 to 2.18 |
| PDA | NIPPV vs NCPAP | 1.01 (0.82 to 1.24) | 0.82 to 1.24 |
| PDA | NHFOV vs NCPAP | 1.05 (0.70 to 1.58) | 0.70 to 1.58 |
| PDA | HFNC vs NCPAP | 0.88 (0.55 to 1.43) | 0.55 to 1.43 |
| NEC | NIPPV vs NCPAP | 0.60 (0.37 to 0.97) | 0.37 to 0.97 |
| NEC | NHFOV vs NCPAP | 0.55 (0.29 to 1.04) | 0.29 to 1.04 |
| NEC | HFNC vs NCPAP | 1.02 (0.36 to 2.87) | 0.36 to 2.87 |
| IVH | NIPPV vs NCPAP | 1.60 (0.75 to 3.43) | 0.53 to 4.87 |
| IVH | NHFOV vs NCPAP | 0.82 (0.31 to 2.22) | 0.23 to 2.96 |
| IVH | HFNC vs NCPAP | 1.66 (0.46 to 6.00) | 0.37 to 7.57 |
| Air leak | NIPPV vs NCPAP | 1.01 (0.37 to 2.76) | 0.29 to 3.53 |
| Air leak | NHFOV vs NCPAP | 0.71 (0.15 to 3.46) | 0.12 to 4.09 |
| Air leak | HFNC vs NCPAP | 0.61 (0.12 to 3.19) | 0.10 to 3.75 |
| Air leak | NIV-NAVA vs NCPAP | 3.00 (0.12 to 72.03) | 0.11 to 78.53 |

Intervals are plug-in normal approximations, calculated from the independent model. When τ² is estimated as zero, they coincide with the CIs. This does not mean that uncertainty about future effects is absent: uncertainty in τ² is not included.

# Table A6 Reconciliation with the reported league tables

| **Outcome** | **Comparison** | **Reported league table** | **Independent REML audit** |
| --- | --- | --- | --- |
| Air leak | HFNC vs NHFOV | 0.86 (0.10 to 7.30) | 0.86 (0.10 to 7.19) |
| Air leak | HFNC vs NCPAP | 0.61 (0.12 to 3.22) | 0.61 (0.12 to 3.19) |
| Air leak | NHFOV vs NCPAP | 0.71 (0.15 to 3.47) | 0.71 (0.15 to 3.46) |
| Air leak | NHFOV vs NIPPV | 0.70 (0.13 to 3.80) | 0.70 (0.13 to 3.76) |
| Air leak | NCPAP vs NIPPV | 0.99 (0.36 to 2.72) | 0.99 (0.36 to 2.71) |
| Air leak | HFNC vs NIV-NAVA | 0.20 (0.01 to 7.38) | 0.20 (0.01 to 7.35) |
| Air leak | NHFOV vs NIV-NAVA | 0.24 (0.01 to 8.27) | 0.24 (0.01 to 8.26) |
| Air leak | NIPPV vs NIV-NAVA | 0.34 (0.01 to 9.45) | 0.34 (0.01 to 9.44) |
| IVH | NHFOV vs NCPAP | 0.82 (0.29 to 2.32) | 0.82 (0.31 to 2.22) |
| IVH | NHFOV vs HFNC | 0.50 (0.08 to 2.96) | 0.50 (0.10 to 2.38) |
| IVH | NCPAP vs HFNC | 0.60 (0.15 to 2.44) | 0.60 (0.17 to 2.17) |
| IVH | NHFOV vs NIPPV | 0.51 (0.13 to 2.00) | 0.51 (0.15 to 1.72) |
| IVH | NCPAP vs NIPPV | 0.62 (0.27 to 1.43) | 0.62 (0.29 to 1.34) |
| IVH | HFNC vs NIPPV | 1.04 (0.28 to 3.84) | 1.04 (0.29 to 3.76) |
| Mortality | NIPPV vs HFNC | 0.87 (0.16 to 4.79) | 0.90 (0.15 to 5.57) |
| Mortality | NHFOV vs HFNC | 0.92 (0.17 to 5.01) | 0.96 (0.16 to 5.83) |
| Mortality | HFNC vs NCPAP | 0.80 (0.18 to 3.60) | 0.77 (0.15 to 3.91) |
| BPD | NHFOV vs HFNC | 0.88 (0.39 to 2.01) | 0.88 (0.40 to 1.97) |
| BPD | NHFOV vs NIPPV | 0.87 (0.40 to 1.86) | 0.87 (0.42 to 1.76) |
| BPD | HFNC vs NIPPV | 0.98 (0.50 to 1.91) | 0.98 (0.50 to 1.90) |
| BPD | NHFOV vs NCPAP | 0.72 (0.37 to 1.41) | 0.72 (0.38 to 1.40) |
| BPD | NIPPV vs NCPAP | 0.84 (0.53 to 1.32) | 0.84 (0.54 to 1.29) |

# References and reproducibility materials

Hutton B, Salanti G, Caldwell DM, et al. The PRISMA extension statement for reporting of systematic reviews incorporating network meta-analyses of health care interventions: checklist and explanations. Ann Intern Med. 2015;162:777–784. doi:10.7326/M14-2385.

White IR. Network meta-analysis. Stata J. 2015;15:951–985. doi:10.1177/1536867X1501500403. This reference identifies a Stata implementation; it does not establish that the supplied manuscript used that package.

Chaimani A, Caldwell DM, Li T, Higgins JPT, Salanti G. Chapter 11: Undertaking network meta-analyses. In: Cochrane Handbook for Systematic Reviews of Interventions, version 6.5. Cochrane; 2024. https://www.cochrane.org/authors/handbooks-and-manuals/handbook/current/chapter-11

van Valkenhoef G, Dias S, Ades AE, Welton NJ. Automated generation of node-splitting models for assessment of inconsistency in network meta-analysis. Res Synth Methods. 2016;7:80–93. doi:10.1002/jrsm.1167. The present independent audit uses conservative disjoint-trial checks, not this paper’s Bayesian implementation.

The reproducibility archive contains arm_data.csv, reported_estimates.csv, analyze.py, audit_results.json, and tabular outputs. Running analyze.py regenerates the numerical audit from the supplied counts. Source IDs 16 to 24 are local audit identifiers and are not final manuscript reference numbers. No patient-level data, unpublished subgroup estimates, or missing clinical characteristics were imputed.

The manuscript’s exact Stata command/package and version, model specification, estimation options, and saved log remain necessary to reproduce its original main results. Final manuscript estimates, rankings, diagnostics, and certainty judgments should be drawn from a single reconciled analysis.
