# Supplementary material for: Comparative efficacy and safety of noninvasive respiratory support in very preterm infants (<32 weeks' gestation): a network meta-analysis
Source: Front Pediatr. 2026 Oct 28;14:1928134. doi: 10.3389/fped.2026.1928134 (PMC13752198; doi:10.3389/fped.2026.1928134)
Supplement: Supplementary file 1 [file Datasheet1.zip › Supplementary material/Supplementary.Funnel plot/funnel plot-NEC.pdf]

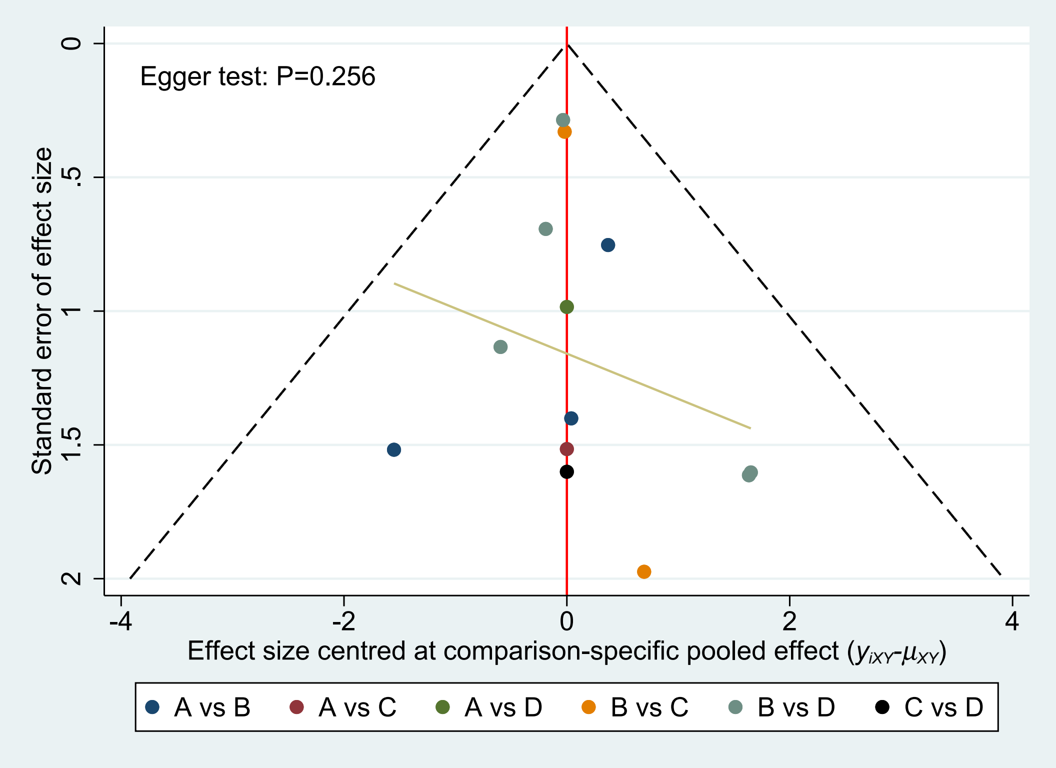

Egger test: P=0.256
Standard error of effect size
0
.5
1
1.5
2
-4
-2
0
2
4
Effect size centred at comparison-specific pooled effect (y_{iXY}-μ_{XY})
A vs B
A vs C
A vs D
B vs C
B vs D
C vs D
