# Supplementary material for: Comparative efficacy and safety of noninvasive respiratory support in very preterm infants (<32 weeks' gestation): a network meta-analysis
Source: Front Pediatr. 2026 Oct 28;14:1928134. doi: 10.3389/fped.2026.1928134 (PMC13752198; doi:10.3389/fped.2026.1928134)
Supplement: Supplementary file 1 [file Datasheet1.zip › Supplementary material/Supplementary.SUCRA/SUCRA-mortality rate.pdf]

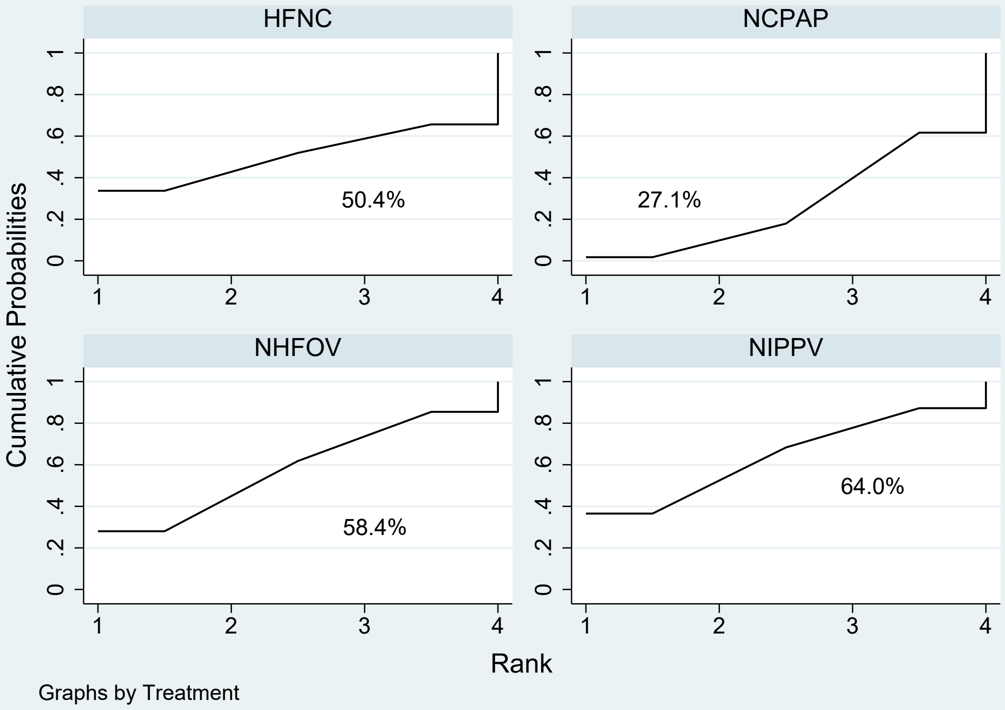
HFNC
NCPAP
NHFOV
NIPPV
50.4%
27.1%
58.4%
64.0%
Cumulative Probabilities
Rank
0
.2
.4
.6
.8
1
1
2
3
4
Graphs by Treatment
